# Immune cell gene expression signatures in diffuse glioma are associated with IDH mutation status, patient outcome and malignant cell state, and highlight the importance of specific cell subsets in glioma biology

**DOI:** 10.1186/s40478-022-01323-w

**Published:** 2022-02-10

**Authors:** Bharati Mehani, Saleembhasha Asanigari, Hye-Jung Chung, Karen Dazelle, Arashdeep Singh, Sridhar Hannenhalli, Kenneth Aldape

**Affiliations:** 1 Laboratory of Pathology, Center for Cancer Research, National Cancer Institute, National Institutes of Health, Bethesda, MD 20892 USA; 2 Cancer Data Science Laboratory, Center for Cancer Research, National Cancer Institute, National Institutes of Health, Bethesda, MD USA

**Keywords:** Glioma, Tumor microenvironment, Malignant cell-state, Deconvolution, CIBERSORTx, Prognosis

## Abstract

The tumor micro-environment (TME) plays an important role in various cancers, including gliomas. We estimated immune cell type-specific gene expression profiles in 3 large clinically annotated glioma datasets using CIBERSORTx and LM22/LM10 blood-based immune signatures and found that the proportions and estimated gene expression patterns of specific immune cells significantly varied according to IDH mutation status. When IDH-WT and IDH-MUT tumors were considered separately, cluster-of-cluster analyses of immune cell gene expression identified groups with distinct survival outcomes. We confirmed and extended these findings by applying a signature matrix derived from single-cell RNA-sequencing data derived from 19 glioma tumor samples to the bulk profiling data, validating findings from the LM22/LM10 results. To link immune cell signatures with outcomes in checkpoint therapy, we then showed a significant association of monocytic lineage cell gene expression clusters with patient survival and with mesenchymal gene expression scores. Integrating immune cell-based gene expression with previously described malignant cell states in glioma demonstrated that macrophage M0 abundance significantly correlated with mesenchymal state in IDH-WT gliomas, with evidence of a previously implicated role of the Oncostatin-M receptor and macrophages in the mesenchymal state. Among IDH-WT tumors that were enriched for the mesenchymal cell state, the estimated M0 macrophage expression signature coordinately also trended to a mesenchymal signature. We also examined IDH-MUT tumors stratified by 1p/19q status, showing that a mesenchymal gene expression signature the M0 macrophage fraction was enriched in IDH-MUT, non-codeleted tumors. Overall, these results highlight the biological and clinical significance of the immune cell environment related to IDH mutation status, patient prognosis and the mesenchymal state in diffuse gliomas.

**Supplementary Information:**

The online version contains supplementary material available at 10.1186/s40478-022-01323-w.

## Introduction

Gliomas are most aggressive and most common primary brain tumors that account for about 80% of all brain malignancies and include lower-grade gliomas (LGG) and glioblastomas (GBM) [46]. LGG typically range from grade 2 to 3 and have better survival, while GBM are categorized as high-grade tumor (grade 4) with poor survival [46]. Previous studies have highlighted the tumor heterogeneity within both LGG and GBM, where LGG were classified into three subtypes: IDH gene mutation with the chromosome 1p/19q codeletion, IDH mutation without 1p/19q codeletion, and IDH wildtype [7], while GBM were categorized into four molecular subtypes: proneural, neural, classical, and mesenchymal [64]. Recently, in the new classification by the World Health Organization (WHO), diffuse gliomas in adults can broadly be divided into three molecular subtypes based on their IDH mutation and 1p/19q codeletion status: (i) IDH wildtype glioma (GBM), (ii) IDH-mutant glioma without 1p/19q codeletion (IDH-mutant astrocytoma), and (iii) IDH mutant glioma with 1p/19q codeletion (IDH-mutant and 1p/19q co-deleted oligodendroglioma) [39].

Current treatment mainly involves neurosurgical resection followed by radiotherapy and temozolomide (TMZ) chemotherapy. Despite such therapies, GBM remains an incurable tumor with a median survival of only 15 months [68]. Recently, different immunotherapeutic strategies have been explored [14], such as immune checkpoint inhibitors and chimeric antigen receptor T cells used in several extracranial cancers [5, 18, 19]. Prior literature suggests that histopathologically similar tumors can respond differently to specific treatments [1, 11, 42]. To account for this heterogeneity, the tumor microenvironment (TME) can be crucial to select the immunotherapy responses [60]. Thus, the complex interplay between tumor cells and their tumor immune microenvironment can influence the outcome of immunotherapy and many other anti-cancer therapies to stratify patients [12, 19, 33]. Recent studies have noticed subtype-specific enrichment of immune cells [61] and their prognostic association in multiple tumors [4, 27, 28, 48, 74]. For example, higher levels of immune cell infiltration are associated with HER-2 positive and triple-negative breast cancers [18, 30]. The immunological heterogeneity of gliomas has also been investigated, revealing the predominant antitumor immune response in the mesenchymal subtype of GBM [13] and identification of immune-specific subtypes in diffuse LGG [72]. Recently, a comprehensive study has analyzed the tumor microenvironment of the brain and demonstrated significant enrichment of tumor-associated macrophages (TAMs) between glioma subtypes and brain metastasis [34]. Additional work has highlighted the role of macrophages in inducing mesenchymal-like state in glioblastoma, showing that macrophage-derived oncostatin M interacts with its receptor on glioma cells which in turn promotes mesenchymal state via STAT3 signaling [24]. Such complex interplay between tumor cells and the tumor immune microenvironment in glioma is therefore worthy of further exploration where deconvolution of bulk expression data in clinically annotated datasets could yield additional insights into this biology as well as its clinical relevance.

Deconvolution of gene expression data is an increasingly used tool to estimate immune cell type proportions, as well as immune cell specific gene expression profiles. CIBERSORTx is an analytical tool that has been proposed to impute gene expression profile from bulk data, providing an estimation of the abundances of member cell types in a mixed cell population [45, 57]. The LM22 and LM10 signature matrices is commonly used to estimate cell type abundance and gene expression of 22 and 10 immune cell types, respectively. Using this approach, we hypothesized that the glioma subtypes classified based on their IDH mutation and 1p/19q codeletion status may harbor distinct tumor immune microenvironments and that specific microenvironmental signatures would correlate to patient outcome within IDH-WT and IDH-MUT gliomas. We also generate a single cell RNAseq dataset of 19 glioma samples to generate a signature matrix highly relevant for deconvolution of bulk glioma expression data to extend key findings from the LM10 and LM22 signatures. To fully explore and evaluate these hypotheses, we use 3 independent glioma datasets (The Cancer Genome Atlas (TCGA) and two Chinese Glioma Genome Atlas (CGGA) datasets) that offered the opportunity for reproducibility and validation of findings. In addition to demonstrating the subtype specific prognostic impact of infiltrating immune cells and their association with the tumor component, our study uncovered a comprehensive framework for future studies designed to characterize the interplay between tumor cells and their surrounding immune cells.

## Methods

### Glioma datasets and immune cell identification

We downloaded publicly available transcriptomic datasets for gliomas from two independent resources: TCGA data portal and Chinese Glioma Genome Atlas (CGGA). From TCGA, we considered LGG and GBM while from CGGA, CGGA325 [3, 75], and CGGA693 [38, 67] were used. These encompass a total of 1721 high to low grade gliomas. Read count matrices from TCGA were normalized to TPM (Transcripts Per Kilobase Million) using a shiny R based COEX-seq package (https://github.com/kimsc77/COEX-seq) [32], while the CGGA datasets were already processed and ready to use for the downstream analysis. These bulk datasets were then subjected to deconvolution against a signation matrix by using CIBERSORTx with its default parameters and B-mode batch-correction [57]. For this study, we employed LM22 and LM10 signatures, where LM22 encompass signatures from 22 individual immune cell types while LM10 is a condensed matrix comprising of 10 broad immune cell types from LM22. Notably, In case of LM10, monocyte includes M0, M1 and M2 macrophages into a single unit while T-CD4 includes T-naïve, T-mem-activ, T-mem-rest, T-gam-del, T-help and T-reg cells. We also included an additional checkpoint inhibitor treated glioma dataset encompassing 29 tumors (GSE121810) and was deconvolved with LM10 signatures due to its small sample size.

### Tumor cell state deconvolution

To deconvolve previously desctibed tumor cell states we used publicly available scRNA Seq datasets from GSE70630 and GSE89567 for IDH-MUT samples, and from GSM3828672 for IDH-WT samples. The cells were labeled by using a previously published method https://github.com/jlaffy/scalop with its scalop::sigScores and scalop::maxcol_strict functions to score cells using previously defined meta-modules. Cells from IDH-WT tumors were processed with MES-like, NPC-like, AC-like and OPC-like meta-modules [44], while those from IDH-MUT were processed with Oligo-like, Astro-like and Stem-like specific genes [63]. Cell having a minimum score difference of 0.3 between the two maximum scoring modules were selected to generate signature matrix by using CIBERSORTx [57]. These malignant cell state signatures from both IDH-WT and IDH-MUT tumors were then independently used to deconvolve the 3 bulk transcriptomic datasets with the default parameters and B-mode batch-correction.

### Cell type-specific gene expression profiling

Unsupervised clustering of gene expression deconvolution data was performed by Seurat v3. For the dimensionality reduction, we used most variable 3000 genes with “Seurat::RunUMAP” function and identified cell type specific clusters [23]. We also explored subclusters of each cell types by using K-means clustering with nstart = 25, iter.max = 500, algorithm = "Hartigan-Wong" and silhouette method to compute the optimal number of clusters. These were visualized by using R based Rtsne package and ggplot function.

### Identifying closely related clusters of cell type clusters

To identify similarly behaving clusters from all the cell types or cell states based on their sample memberships we employed a binary approach, where "1" denotes the presence of a sample in a cluster while "0" represents its absence. These clusters were then subjected to unsupervised clustering by using a R based ConsensusClusterPlus package with distance = pearson and maxK = 6, reps = 50 [70]. We used NbClust R package to determine the optimal number of clusters and the results were visualized by using a R based ComplexHeatmap package [20].

### Functional annotation of the identified clusters

We used R based singscore package to score each sample against an immune signature, pathways, or biological processes [15]. The hallmark epithelial-mesenchymal transition (EMT) gene-set used in the analysis were downloaded from Molecular Signatures Database (MSigDB) [37]. We obtained Cytolytic (CYT) score by calculating the geometric mean of GZMA and PRF1 expression [51] in each dataset from each of the identified clusters. Similarly, T cell exhaustion (TCE) score was calculated by using PD-1, CTLA-4, TIM-3, LAG-3, CD160, 2B4, TIGIT, CD39, and BTLA genes [9]. We also attempted to estimate the STAT3-based gene scores by using STAT3, ELK3, DTX3L, AIDA, NEDD9, KLHDC8A, TWSG1, NAGA, MYO1C, SH3PXD2B, SLC35F5, HERC5, C5orf15, ZMYM6, TPM4, DAP, SNAP23, RHOJ, HMG20B, ZCCHC9, NAMPT, SLC43A3, BIRC2, BACE2, ITGB1, ITFG3, AGXT2L2, GNG12, PALLD, IGF2BP2, NUP37, CTNNA1, GMPPA, BRCA1, TMEM51, RPN2, FZD1, PTPN12, SHQ1, and NAA38 genes [58]. We used GENECODIS 4.0 beta for gene set enrichment analysis for the prognostic genes derived from M0 macrophages [8].

### Survival analysis and statistical testing

Kaplan–Meier survival curves were computed by a R based “survival::survfit” function and visualized by “survminer::ggsurvplot” function. Multivariate Cox-regression was performed by “survival::coxph" function. To examine the statistical significance between the groups we either used chi-Square test for 2X2 matrix or Fisher’s exact test for other combinations. The evaluated significance levels were denoted by *** when p values < 0.0001; by ** when p values < 0.001; or by * when p values < 0.05. The overall workflow followed in this article is described in Additional file 1: Figure S1.

### Single cell RNA-sequencing and data processing

Fresh tumor tissues were processed using Brain Tumor Dissociation Kit (P) and gentleMACS Dissociator (Miltenyi Biotec) following manufacturer’s protocol. Erythrocytes were removed using a density gradient separation medium (Lympholyte-H; Cedarlane Laboratories) and resulting cell suspensions were cleared from tissue debris using Debris Removal Solution (Miltenyi Biotec). Cells were counted and assessed with Luna-FL Automated Cell Counter (Logos Biosystems) and run on 10 × Chromium Next GEM Chip with target recovery of 6,000 cells per lane. Library generation, quality control, and sequencing were all performed following 10 × Genomics Single Cell 3’ Reagent Kit User Guide.

Read demultiplexing and alignment to the GRCh38 human reference genome was performed using the Cell Ranger Single Cell Software v2.0 (10× Genomics) with the cellranger’s mkfastq and count functions, respectively. Raw count matrices were filtered for the minimum number of genes detected per cell (greater than 200) and the percentage of mitochondrial unique molecular identifier (UMI) counts (less than 20%). Filtered barcodes were then merged and clustered using the Seurat v3 package in R as per the developers’ vignettes. Cell annotation and malignant cells identification were performed using scmap and CONICsmat. These annotated cells were used to generate signature matrix using CIBERSORTx with its default parameters and S-mode batch-correction.

## Results

### M2 macrophage abundance varies across glioma subtypes and correlates with patient survival

**Fig. 1:**
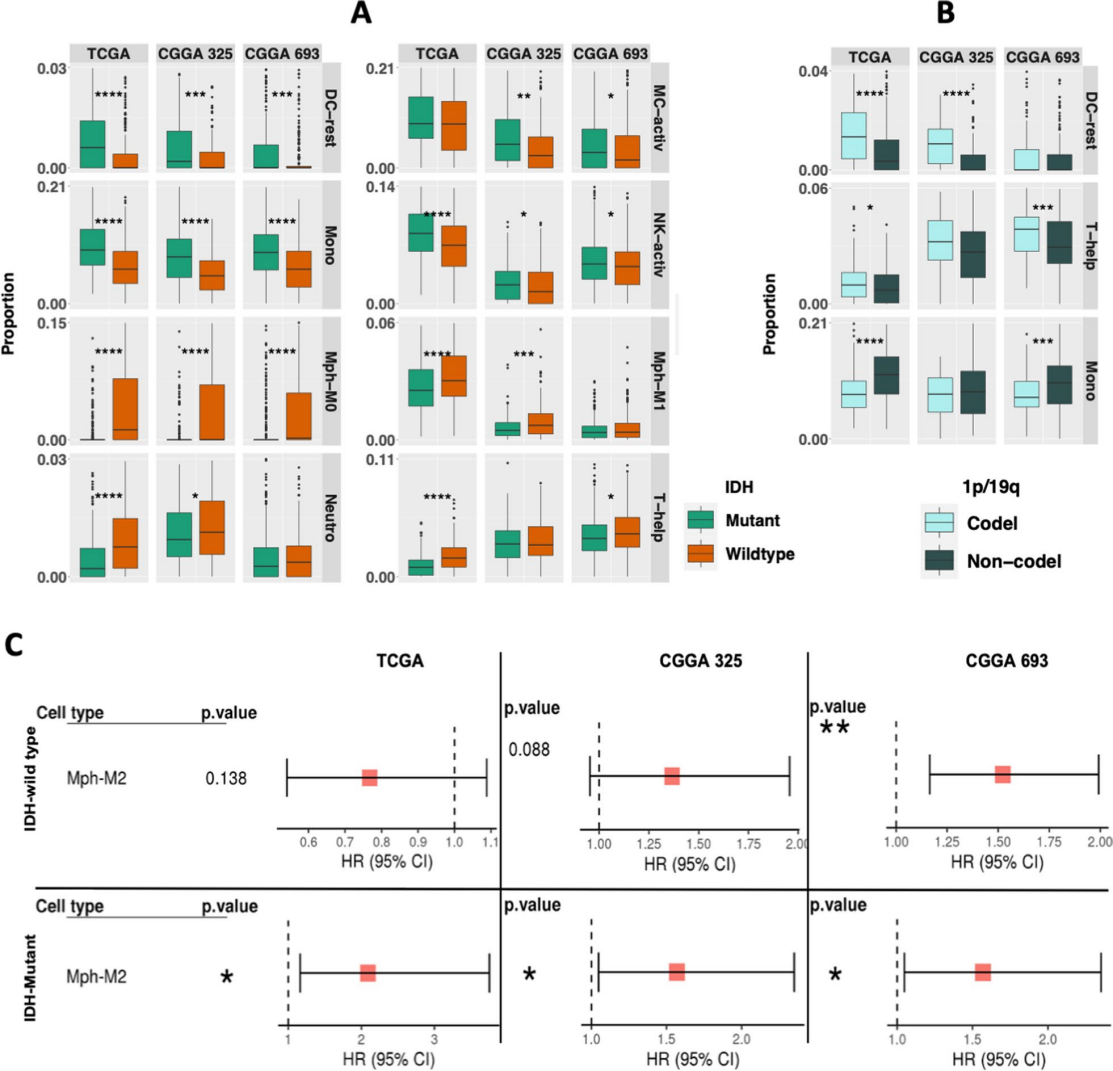
An overview of the infiltrated immune cells in glioma and its subtypes. **A** Differentially represented cell types between IDH-MUT (green) and IDH-WT (orange) tumors are depicted by using boxplots. Similarly, **B** boxplots representing relative abundance of the cell types between Chr1p/19q codeleted (cyan) and non-codeleted (dark green) samples in IDH-MUT tumors. **C** Forest plots displaying prognostic association of M2 macrophages in both IDH-WT and IDH-MUT tumors. The abbreviations used for each cell type is described in Additional file 13: Table_S1

We first examined the composition of infiltrating immune cells in gliomas, we utilized CIBERSORTx [45] on 3 diffuse glioma datasets (TCGA, CGGA325 and CGGA693), using the validated LM22 signature matrix (Additional file 13: Table S1 and Additional file 2: Figure S2A). Similar to previous reports, we also found M2 macrophages were the most abundant cell type across all IDH mutant (IDH-MUT) and wildtype (IDH-WT) tumors [61, 65]. We also observed that specific immune cell distributions were associated with IDH status. For example, M0 and M1 macrophages, neutrophils and T-helper cells were more abundant in IDH-WT gliomas, while monocytes, resting dendritic cells, activated mast cells, and activated natural killer cells were significantly enriched in IDH-MUT tumors (Fig. 1A). However, within IDH-MUT tumors, monocytes were more represented in the non-codeleted tumors, while resting dendritic cells and T-helper cells were abundant in the1p/19q codeleted tumors (Fig. 1B). These findings are in line with previous studies [34, 43, 49, 69]. Next, we evaluated the association of tumor-infiltrating immune cell fractions with survival outcomes. Survival plots revealed that high proportion of M2 macrophages can predict poor prognosis across all datasets in both IDH wildtype (except TCGA) and mutant tumors (Fig. 1C). Previous reports have highlighted an association between immune infiltrate and DNA damage [61]. In line, we also found positive correlations for M2 macrophage abundance with various genomic alterations like number of segments, fraction of genome altered, and homologous recombination defects in TCGA datasets, where ‘‘fraction altered’’ represents the fraction of bases deviating from baseline ploidy (defined as above 0.1 or below -0.1 in log2 relative copy number (CN) space), while ‘‘number of segments’’ represents total number of segments in each sample’s copy number profile (Additional file 2: Fig. 2B). These results agree with prior studies and suggest altered tumor microenvironments in diffuse gliomas based on IDH-mutation status, as well as correlations of the microenvironment with genomic alterations in the neoplastic cells.

### Immune cell expression clusters are associated with IDH status, tumor grade, and patient survival

**Fig. 2:**
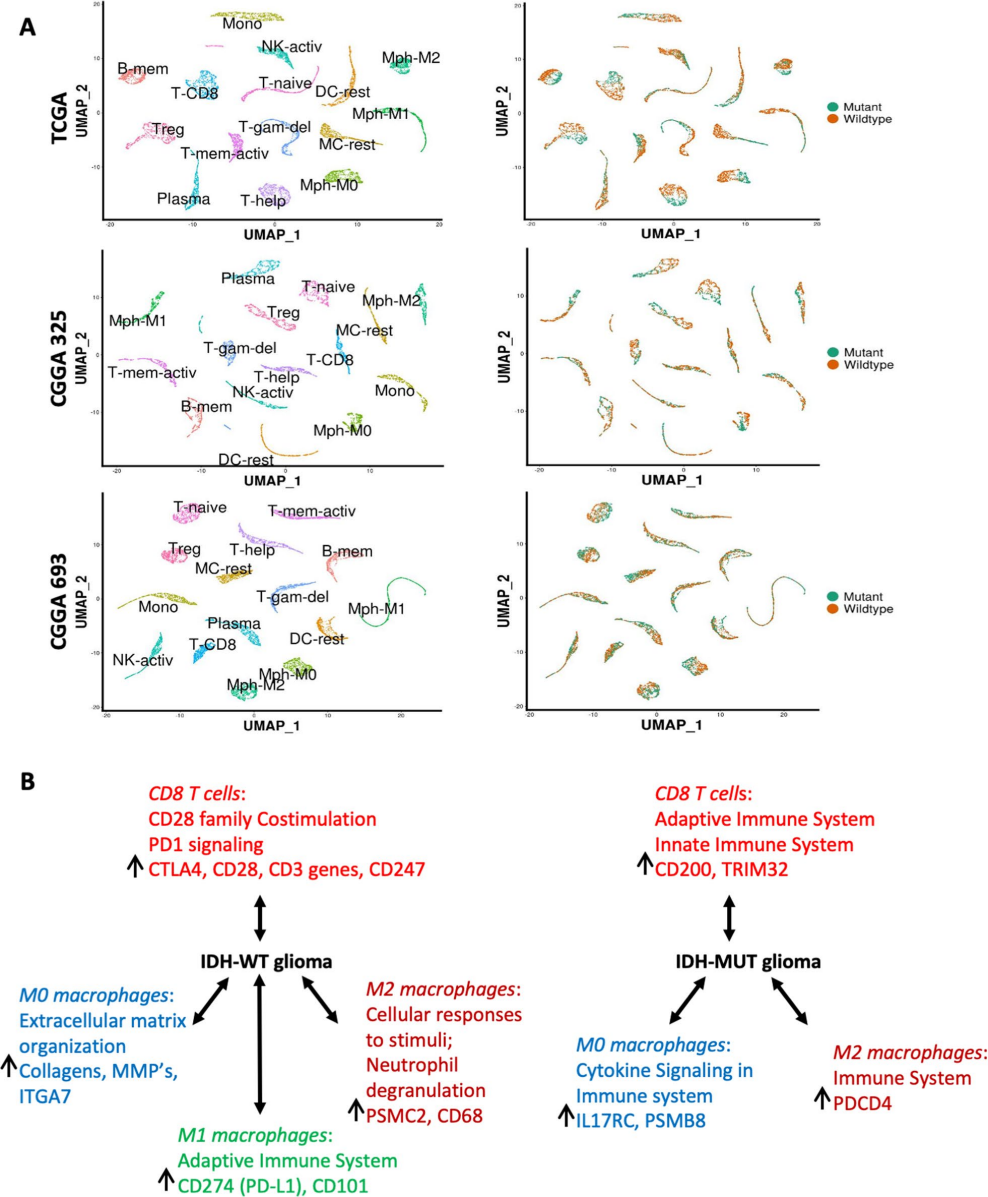
Gene expression clustering and the separation between IDH-MUT and IDH-WT tumors. **A** UMAPs denoting 15 independent gene expression clusters identified in all 3 datasets harboring a clear separation between IDH-MUT (green) and IDH-WT (orange) tumors. **B** Most significant pathways and related genes derived from selected cell types demonstrates comparative transcriptional profiles between IDH-WT and IDH-MUT tumors

In addition to estimating the proportions of individual cell types, CIBERSORTx also estimates the gene expression profile of each cell type in each sample. Figure 2A shows the transcriptomic clustering of 22 cell types across all samples independently in each of the three cohorts, Interestingly, gene expression profiles 15 of the 22 immune cell types revealed a clear separation between IDH mutant and wildtype samples in all 3 datasets (Fig. 2A). We also analyzed expression profiles of the genes associated with macrophages and T cells from IDH-MUT and IDH-WT tumors. This set of analysis highlighted the expression-based cell-type specificity between the two subtypes. For example, in IDH-WT tumors, genes expressed in M0 macrophages were significantly enriched for mesenchymal pathways while in IDH mutants it was immunological pathways (Fig. 2B).

Focusing further on M2 macrophages, we observed fine clusters that distinctly segregate by tumor IDH status across all three datasets (Additional file 2: Fig. S2C and D) and exhibit distinct survival characteristics (Additional file 2: Fig. S2E). Importantly Cox multivariate analyses, after adjusting for relevant covariates (IDH mutation status, tumor grade and patient age) indicated that these unsupervised cluster groups from deconvolved M2 macrophage gene expression data remained independent predictors of patient outcome (Additional file 2: Fig. S2F). Additional analysis using LM10 signatures, (where monocyte includes M0, M1 and M2 macrophages into a single unit while T-CD4 includes T-naïve, T-mem-activ, T-mem-rest, T-gam-del, T-help and T-reg cells) also showed distinct cluster representing each cell type. The majority of these cell types again showed a clear separation between IDH-MUT (green) and IDH-WT (orange) tumors (Additional file 3: Fig. S3A). For example, monocytes showed clean clusters that were distinctly segregated by their IDH status and distinct survival supports our results even with fewer cell types (Additional file 3: Fig. S3B, C and D).

For comparison, we next examined M2 macrophage expression-based clusters in non-glioma TCGA tumors but did not find such significant survival associations in most tumor types (Additional file 4: Figure S4), suggesting that the prognostic association of macrophage-based gene expression groups may be relatively specific to gliomas.

### Tumor groups with distinct immune signature patterns reveal distinct clinical features

**Fig. 3:**
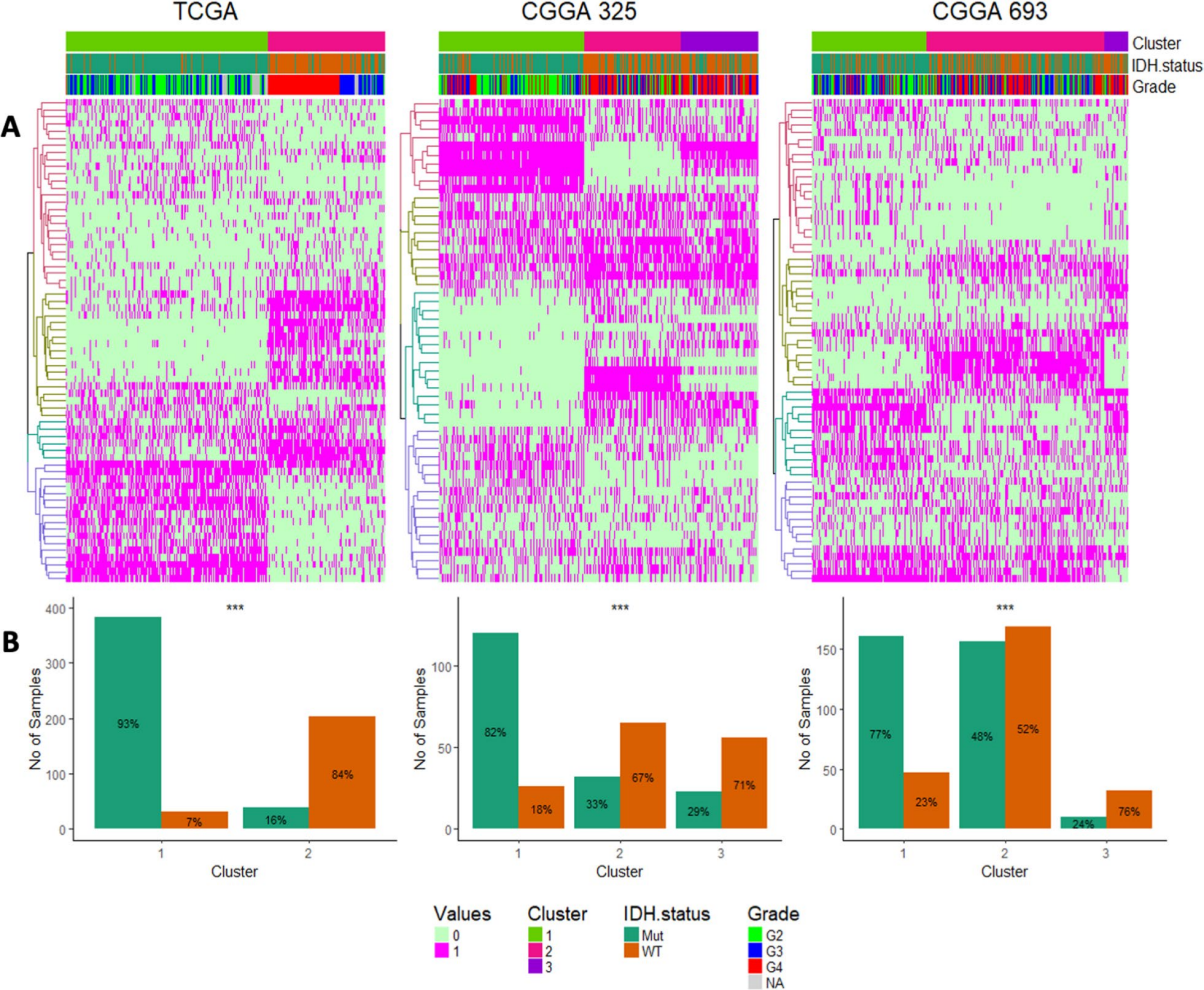
Cluster of cluster analysis of immune based clusters. **A** Heatmaps representing the hierarchical clustering of LM22 based clusters broadly differentiating IDH-WT tumors from IDH-MUT. **B** Histograms showing quantitative distribution IDH specific tumors across each cluster

Based on the above analyses, multiple expression-based clusters were identified for each immune cell type. For instance, there were 3 clusters for M2 macrophages, 2 clusters for regulatory T cells, 4 clusters for monocytes, and so on. The unsupervised (k-means) clustering of each immune cell type-based clusters were next examined together to identify identical groups of immune-based clusters in glioma. Towards that end, we used cluster-of-cluster analysis (COCA), an unsupervised method to integrate multi-omics data into biologically relevant sub-classes [26]. By applying COCA to these immune based clusters for all 22 immune cell types, we found distinct clusters of glioma samples significantly grouped by their IDH status, as shown in Fig. 3A and B, supporting immune cell signatures underlie the major glioma clinical subtypes.

We then separated the samples according to IDH mutation status (e.g., IDH-WT and IDH-MUT samples were analyzed separately) and performed COCA on these tumor groups in all 3 datasets. Among IDH-WT tumors (Additional file 5: Figure S5A), tumor clusters derived from this unsupervised analysis showed significant survival associations across all 3 datasets. Among IDH-MUT tumors (Additional file 5: Figure S5B), 2 of 3 datasets (TCGA and CGGA325) showed significant survival associations among these immune cell-based clusters, while the 3rd dataset (CGGA693) showed a statistical trend. These cluster memberships are listed in Additional file 14: Table S2.

**Fig. 4:**
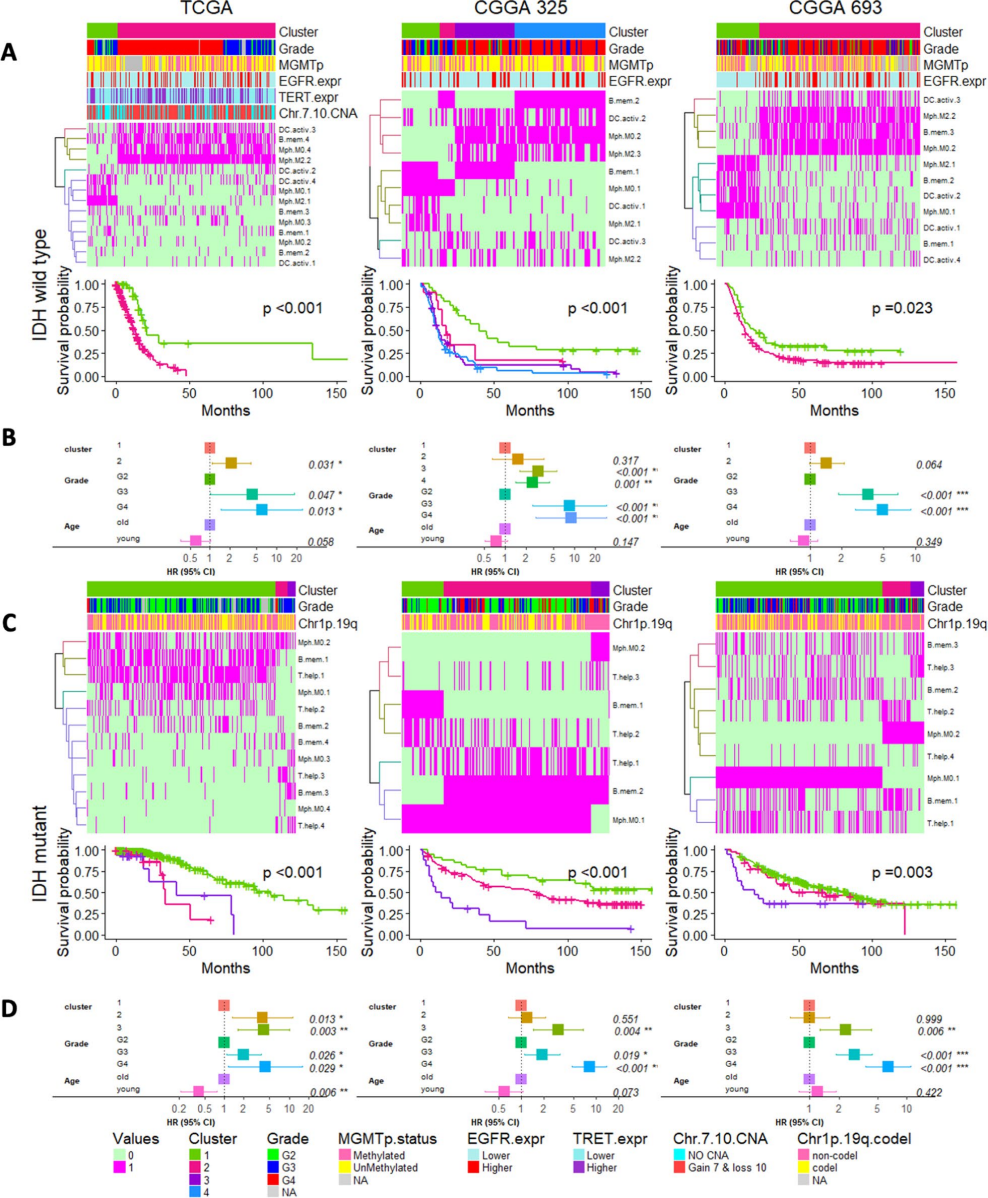
Tumor groups with distinct immune signatures based on selected cell types. **A** Heatmaps representing hierarchical clustering of the clusters identified from selected immune cell types from IDH-WT tumors. The above annotation bars representing the distributions of cluster assignments, tumor grade, MGMT promoter methylation, EGFR expression, TERT expression (surrogating the status of TERT promoter mutation) and Chr7 gain & Chr10 loss followed by their Kaplan–Meier curves denoting their survival differences and **B** forest plots displaying prognostic association of these immune based clusters for IDH-WT. Similarly, **C** heatmaps representing the hierarchical clustering of the clusters identified from selected immune cell types from IDH-MUT tumors. The above annotation bars representing the distributions of cluster assignments, tumor grade and Chr1p/19q loss with Kaplan–Meier curves below the heatmap denoting their survival differences and **D** forest plots displaying prognostic association of these immune based clusters for IDH-MUT tumors

The data represented in Additional file 5: Figure S5, included all 22 cell types and to further characterize important immune cell types, next we focused on those individual cell types whose gene-expression-based cluster groups were prognostic. Within IDH-WT tumors, we tested the gene expression cluster groups from each cell type to identify which ones showed a significant association with patient prognosis (Additional file 6: Figure S6). These included expression clusters derived from M0 macrophages, M2 macrophages, B-mem and DC-active cells were consistently prognostic across all 3 datasets in IDH-WT gliomas (Additional file 6: Figure S6A). Among IDH-MUT tumors, M0 macrophages, T-help and B-mem cell derived cluster groups were found to be consistently prognostic (Additional file 6: Figure S6B). We then applied these respective cluster groups to COCA analyses (Fig. 4) and found that samples defined by these COCA cluster groups remained prognostic in multivariate Cox analyses even after adjusting for clinical variables (patient age and tumor grade) across all 3 datasets for both IDH-WT (Figs. 4A and B) and IDH-MUT tumors (Figs. 4C and D). This results further point to the potential clinical relevance of immune cell biology in predicting patient outcome.

We then tested for macrophage abundance among these immune based clusters. Based on previous reports, macrophages and microglia can be distinguished by ITGA4 expression, where elevated expression in ITGA4 represents macrophage marker [6, 34]. We found increased levels of ITGA4 in poor-surviving groups as compared to cluster groups with better survival across all 3 datasets for IDH-WT tumors and in 2/3 datasets for IDH-MUT tumors (Additional file 7: Figure S7A), suggesting a link between macrophage abundance, immune-based clusters, and the patient outcome in both IDH-WT and IDH mut gliomas.

We next explored cytolytic and T cell exhaustion scores and their survival associations with the identified immune cell-based clusters. With this set of analyses, we observed that higher levels of cytolytic and T cell exhaustion scores among the poor surviving groups (Additional file 7: Figure S7B and C) and are in line with the previous reports [22, 71], further highlighting the biological or clinical significance of tumor microenvironments and their prognosis in gliomas.

### Malignant cell states interact with immune microenvironment signature

**Fig. 5:**
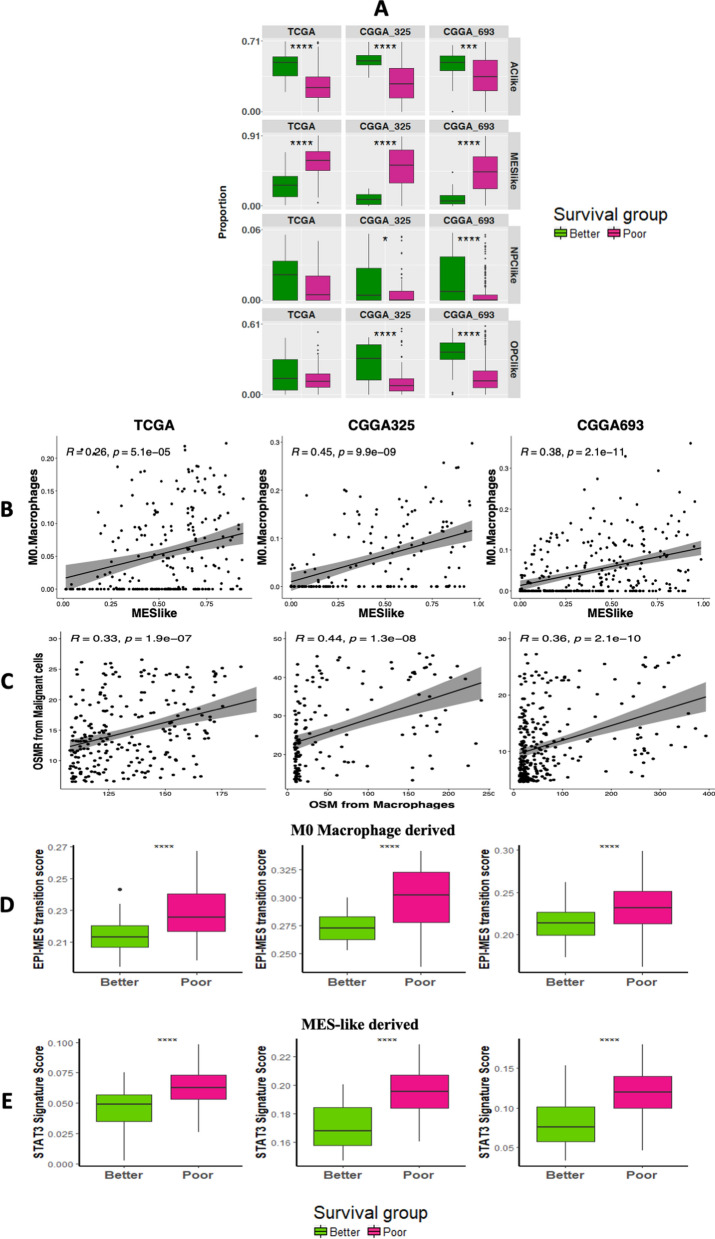
Deconvolving malignant cell states and their interaction with immune based clusters. **A** Differential distributions of IDH-WT specific malignant cell-states between the two immune-based survival groups are depicted by boxplots. **B** Scatter plots representing a positive correlation between the proportions of M0 macrophages and MES-like component of IDH-WT tumors across all 3 datasets. **C** Scatter plots representing a significant positive correlation between the expressions of OSM from macrophages and OSMR from malignant cells in IDH-WT tumors identified by scRNA based deconvolution. **D** Boxplots depicting differential distribution of epithelial-mesenchymal transition markers between the two immune-based survival groups. Similarly, **E** boxplots depicting differential distribution of STAT3 signaling markers between the two immune-based survival groups

To further characterize the role of the TME and their potential interactions with the malignant cell states, we estimated the composition of tumor cell states in all the 3 glioma datasets. To better understand the composition of their malignant cell states we leveraged previously published single-cell resources along with their cell-annotations to derive the signature matrix. As previously established, the diverse malignant cells in IDH-WT tumors have been described as converging to a 4 cell-states: (AClike, MES-like, NPClike and OPClike) [44] while in case of IDH-MUT gliomas, 3 malignant cell-states have been proposed: Astro-like, Oligo-like and Stem-like [63]. Malignant cell-state proportions derived with these signatures were generated using CIBERSORTx and are depicted in Additional file 8: Figure S8A for IDH-WT and Additional file 8: Figure S8B for IDH-MUT tumors, respectively. With the malignant cell state deconvolution, we examined the interplay between these malignant cell-states and immune cell-based (COCA) survival groups. Towards this end, we tested for the distribution of each of the cell states among each of the immune derived survival clusters and found in the case of IDH-WT tumors, the MES-like component was higher in poor surviving groups (as defined in Fig. 4) for all three datasets (Fig. 5A). To further characterize this finding, we built on results from several recent reports which have underlined the role of macrophages to induce MES-like cell-state by implicating macrophage-derived oncostatin M (OSM) with its receptors and activating STAT3 signaling in glioma [24, 47, 66]. Along this line, we found a positive correlation for the proportion of MES-like state and estimated OSMR expression within M0 macrophages (Fig. 5B), but not within M1 macrophages, M2 macrophages or monocytes (Additional file 9: Figure S9), suggesting specificity within macrophage subtypes in a potential interaction with the MES-like malignant cell state. Moreover, the enrichment of an epithelial-mesenchymal transition signature as well as STAT3 signaling markers in poor-surviving groups further characterize these results (Fig. 5D and E), suggesting a role of M0 macrophage abundance in the MES-like cell state in glioma. We also included in-house scRNA RNA seq data from 19 gliomas encompassing 10 IDH-WT and 9 IDH-MUT tumors (Additional file 15: Table S3). With this set of analysis, we were able to identify 18 distinct clusters (Additional file 10: Fig. S10A and B), including the neoplastic and the immune cells (Additional file 10: Fig. S10C, D and Additional file 15: Table S3). These single cells derived cell types were then used to generate the signature matrix for deconvolution (Additional file 10: Fig. S10E and Additional file 15: Table S3). We also assessed the expression deconvolution data from macrophages and malignant cells and found a statistically significant correlation between the expressions of OSM and OSMR expressions from the macrophages and malignant cells respectively (Fig. 5C). This set of analysis support the connection between macrophages and MES-like cells.

Additionally, we identified 368 MES-like corelating genes from M0 macrophages that were common in all 3 datasets that, in turn, showed enrichment for mesenchymal pathways, including extracellular matrix organization and angiogenesis along with the immunological pathways (Additional file 11: Figure S11A). In contrast, 95 genes from M2 macrophages which were common in all 3 datasets showed enrichment only for immunological pathways (Additional file 11: Figure S11B). We next analyzed IDH-MUT tumors and interestingly in 1p/19q non-codeleted tumors (compared to co-deleted tumors) we observed a similar enrichment for genes from mesenchymal pathways in M0 macrophages with overlapping correlated genes compared to the M0 gene expression signatures in the MES-like state of IDH-WT glioma (Additional file 12: Figure S12).

### Extension of findings to checkpoint inhibitor-treated glioma patients

**Fig. 6:**
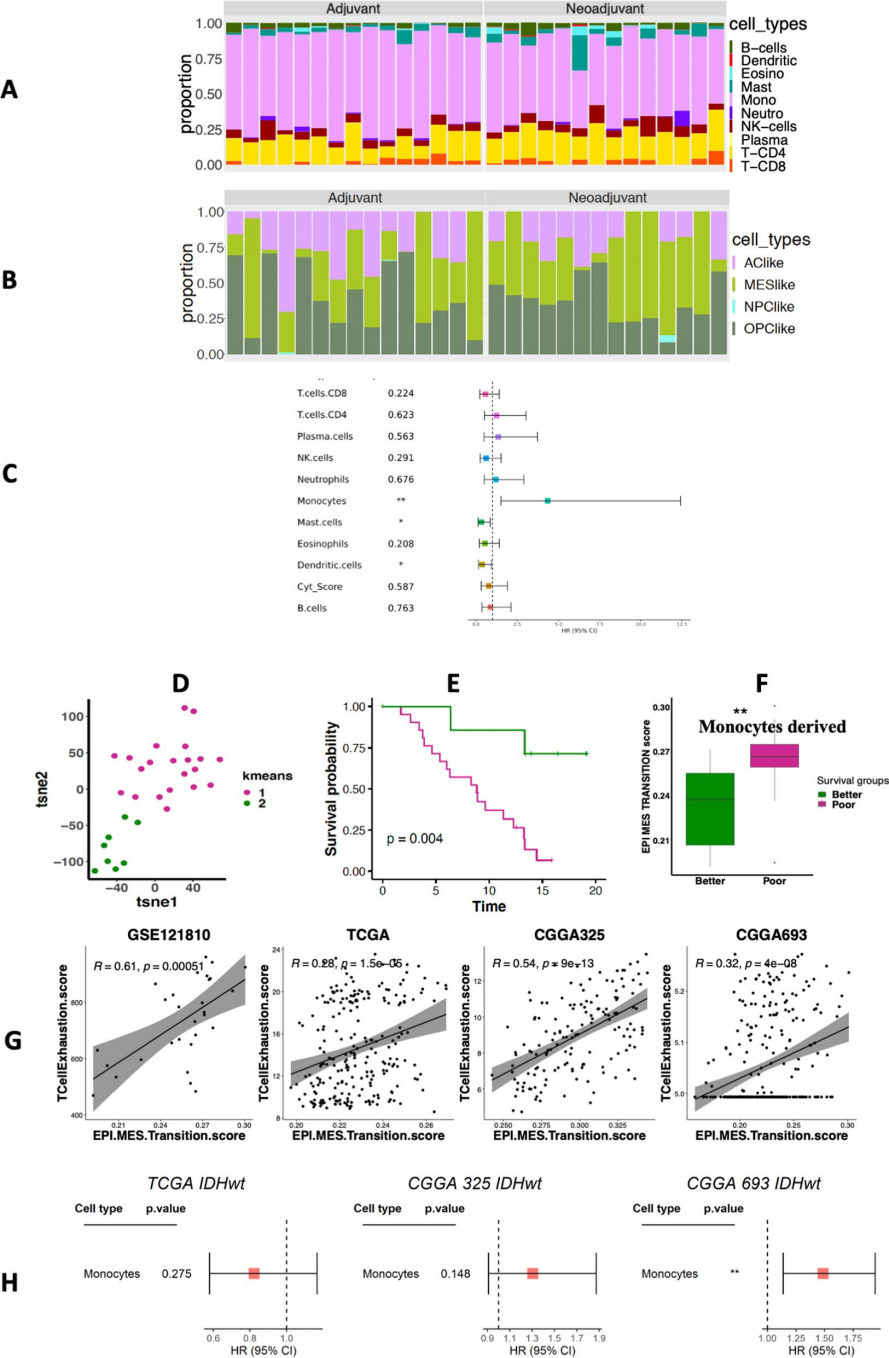
Immunotherapy-treated glioma datasets. **A** Stacked bar plots representing relative proportion of 10 broader category of immune cell types from LM10 across samples treated with checkpoint inhibitor. **B** Stacked bar plots representing the relative proportions of 4 malignant cell-states across all immunotherapy treated samples. **C** Forest plot representing the association between their dichotomous immune cell fractions and their overall survival. **D** Monocyte-based gene expression clusters represented by tSNE followed by **E** Kaplan–Meier curves denoting distinct survival between the two clusters. **F** Boxplots depicting differential distribution of epithelial-mesenchymal transition markers between the two immune-based survival groups derived from monocytes from samples treated with checkpoint inhibitor. **G** Scatter plots representing significant positive correlations between the epithelial-mesenchymal transition and the T cell exhaustion scores both in patients treated with a checkpoint inhibitor, as well as larger cohorts. **H** Forest plot representing the insignificant or inconsistent association between their dichotomous monocytic fractions in non-immunotherapy or non-checkpoint inhibitor treated IDH-WT tumors (from TCGA, CGGA325 and CGGA693) and their overall survival

Next, we investigated infiltrating immune cell compositions in a predominantly IDH-WT 29-sample dataset collected from patients treated with pembrolizumab [10]. To explore the role of tumor-microenvironment in response to immunotherapy, we relied on the LM10 signatures comprising 10 broad immune cell types better suited for smaller datasets than the LM22. Notably, LM10 broadly covers all types of monocyte-derived cells (including M0, M1 and M2 macrophages) in a single unit. As expected from LM10-based deconvolution, this dataset demonstrated enrichment of the monocyte cell fraction across all the patients analyzed (Fig. 6A) and the predicted malignant cell state proportions are shown in Fig. 6B. Interestingly a higher fraction of monocytes was significantly associated with a shorter overall survival (HR = 5.0, P < 0.001) (Fig. 6C), suggesting that cells with monocytic lineage may play a role on checkpoint inhibitor-related patient outcome in glioma. We also repeated the LM10 based deconvolution for TCGA and CGGA datasets and observed higher fraction of monocytes was significantly associated with patient survival only in case of CGGA693 but not in CGGA325 and TCGA tumors (Fig. 6D). Such an inconsistency among the non-immunotherapy treated samples compared to the checkpoint inhibitor-treated tumors suggest the predictive value of monocytes.

Unsupervised clustering of the checkpoint inhibitor-samples based on monocytic expression-based clusters (as above) revealed two clusters with distinct survival outcome (Fig. 6E and F). We also found epithelial-mesenchymal transition (EMT) markers to be significantly enriched in the poor surviving group (Fig. 6G). We also observed a positive correlation between the epithelial-mesenchymal transition and the T cell exhaustion scores both in patients treated with a checkpoint inhibitor, as well as larger cohorts (Fig. 6H). The finding of an association of epithelial-mesenchymal transition with T-cell exhaustion in glioma may be analogous to what has been reported in other cancer types [41]. These results together emphasize prognostic associations of monocyte/macrophage lineage cells in gliomas in the context of patients treated with a checkpoint inhibitor, and link mesenchymal gene expression in monocytic lineage cells with a T-cell exhaustion score in gliomas overall.

## Discussion

Emerging evidence has highlighted the critical involvement of the immune microenvironment in tumor development and progression. These immune-related biomarkers may have a potential to predict patient outcome and therapy responsiveness [2, 31, 54, 73], and thus, have inspired numerous microenvironment-targeted therapies in multiple tumors [33, 34, 50]. In addition, various reports have discussed prognostic roles of immune related genes in gliomas, but the characterization of immune cell compartments has not been fully elucidated.

In this work, we explored the landscape of glioma infiltrating immune cells using CIBERSORTx deconvolution as summarized in Fig. 1. We found M2 macrophages constitute a large portion of glioma microenvironment. Macrophages are a large component of many tumor types and have been an attractive target for glioblastoma therapy [21, 36, 40, 55]. Deconvolution of the tumor bulk transcriptomic data enabled us to investigate immune cell distributions in glioma and their clinical outcome correlated at a much greater resolution. We found that specifically M0 macrophages were enriched in IDH-WT gliomas while monocytes were relatively more abundant in IDH-MUT gliomas. Additionally, higher levels of M2 macrophages were observed to be associated with poor prognosis. These differentially distributed monocyte/macrophage lineage cells and their association with patient outcome further highlight their prognostic roles in tumor progression. Targeting these specific cells to inhibit their tumor-promoting effect and reprogramming them into an anti-tumor phenotype could be a potential therapeutic approach for glioma.

Gene expression profiling of all the deconvolved immune cell types resulted into sample groups segregated by their IDH status, as discussed in Figs. 2 and 3. Further clustering of these sample groups resulted into distinct clusters which can predict patient outcome. These immune based clusters were broadly characterized by tumor grades, and in case of IDH-MUT tumors, also by their 1p/19q codeletion status. Based on WHO 2021 classification and other previous reports has already established that Chr7 gain & Chr10 loss, TERT mutation along with EGFR expression and MGMT promoter methylation are the strongest predictor of survival in glioblastomas [25, 35, 39]. Several reports suggest elevated levels of ITGA4 denoting macrophage abundance can also predict poor prognosis in gliomas [6, 34, 56]. We also observed similar associations between the Chr7 gain & Chr10 loss, expressions of EGFR, TERT (surrogating the status of TERT promoter mutation) and MGMT promoter methylation of the immune based clusters with the patient outcome (Fig. 4A and Additional file 5: Figure S5A). Similarly, the prognostic nature of these immune based clusters was also characterized by higher levels of ITGA4 expression as well as higher scores for cytolytic and T cell exhaustion, as reported previously [22, 51, 71] (Additional file 6: Figure S6), providing corroboration that CIBERSORTx-based deconvolution of the bulk datasets yields result that are consistent with prior investigations. These findings indicate that tumor microenvironment plays a major role in characterizing tumor subtypes [29, 53, 59]. These identified sample groups may exhibit a distinct immune context, prognosis, and immunotherapy benefit, which supports the idea that the immune environment is of vital importance in predicting patient prognosis and evaluating the response rate of checkpoint inhibitor immunotherapies. Later, clusters derived from a few selected cell types that involve M0 and M2 macrophages along with B-mem, DC-activ and T-help, were able to identify tumor subgroups that can also predict patient outcome. This set of analysis again emphasizes the prognostic role of M0 and M2 macrophages along with other immune cells. Further analyses may facilitate more opportunities to investigate other immune cells playing important roles in complex tissues.

**Fig. 7:**
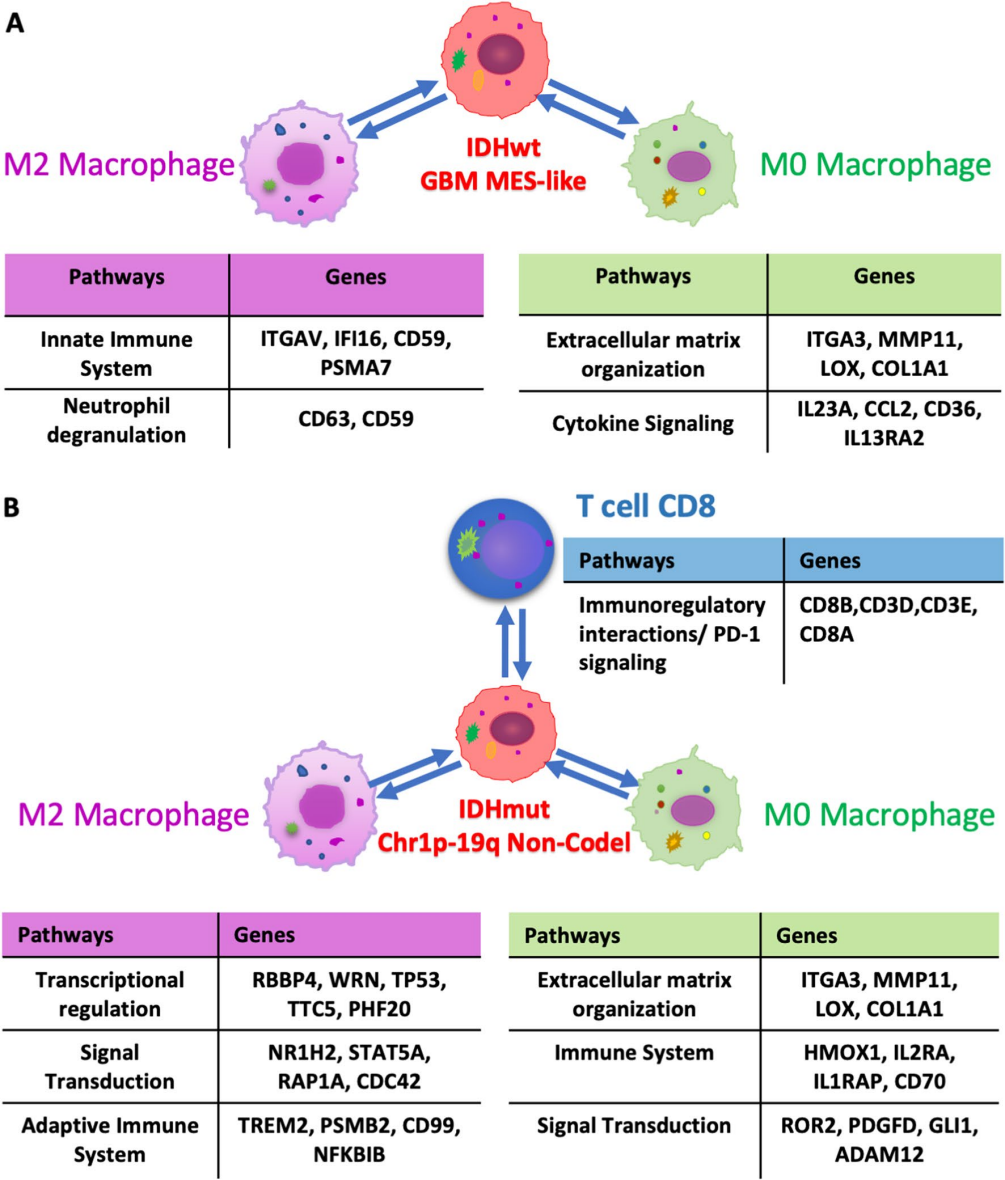
Enrichment for genes from mesenchymal pathways in M0 macrophages while M2 macrophages demonstrated the enrichment for immunological pathways in **A** IDH-WT and **B** IDH-MUT tumors

Recent advances in tumor biology have revealed interactions between the tumor cells and their adjacent microenvironment, highlighting the importance of TME and their involvement in tumor growth and the development of metastasis [52, 62]. Towards this we extended our search by analyzing the proportion of malignant cell-states by using a previously published single cell data and their labels to derive a new signature matrix [44, 63]. To study the interplay of malignant cell-states and immune cell-based clusters, we checked for the distribution of malignant-states among the immune based clusters. With this set of analysis, we found in IDH-WT, MES-like component was found to be higher in poor surviving groups while in IDH-MUT, Oligo-like tumor cell-state was found to be higher in better surviving group (Additional file 7: Figure S7C). Based on recent literature, proportion of TCGA-MES subtype can be correlated with macrophage abundance [24, 66]. On the similar note, in IDH-WT we also found positive correlation between the M0 macrophages and MES-like proportions (r = 0.26, 0.45, and 0.38 in TCGA, CGGA325, and CGGA693 respectively in Fig. 5B) followed by enrichment for genes from mesenchymal pathways along with the immunological pathways in M0 macrophages while M2 macrophages demonstrated the enrichment only for immunological pathways. Together these results highlight the specificity within macrophage subtypes in context to the MES-like cell state in gliomas, as described in Additional file 9: Figure S9 and summarized in Fig. 7A. Additionally, IDH mut tumor also harbor a similar enrichment for genes from mesenchymal pathways in M0 macrophages over other cell types when comparing 1p/19q non-codeleted tumors with codeleted tumors (in Fig. 7B and Additional file 10: Figure S10). A deeper understanding of interactions like this will help not only to explore the cancer growth and metastasis but also to elucidate the mechanisms of action of classical drugs that have been discovered by empirical approaches. Such studies may highlight the importance of deconvolution approaches that can facilitate designing and development of novel cancer drugs.

We expanded our analysis our findings in an independent checkpoint inhibitor-treated glioma samples encompassing 29 high-grade glioma patients treated with pembrolizumab [10]. Here due to the small sample size we used LM10 signature matrix instead of LM22 for deconvolution, where monocytes are expected to encompass all types of macrophages along with monocytes itself. Results showed monocytes were the most predominant cell type and can predict poor prognosis in this glioma samples treated with checkpoint inhibitor (Fig. 6). Further clustering of the deconvolved gene expression from monocytes resulted into poor and better surviving groups where epithelial-mesenchymal transition markers were found to be enriched in the poor surviving group. As monocytes and macrophages share their lineages [17], these findings are consistent with those observed from TCGA and CGGA datasets. These results underline the prognostic potential of monocyte/macrophage lineage cells in gliomas, including patients treated with a checkpoint inhibitor.

Strengths of our study includes the use of clinically annotated genomically characterized glioma data, with the inclusion of 3 independent datasets, allowing the opportunity to examine reproducibility across cohorts. Our study has several limitations, which includes the use of the LM22 gene signature matrix, which was derived primary from cells in the blood and not from tumors. It is likely that variation in immune gene expression signatures exist within solid tumors. In addition, the distinction of M0, M1 and M2 macrophages may not best delineate the function of these cells in gliomas. That said, a recent report indicates that the M0 (unpolarized) state may best represent the majority of macrophages in gliomas [16]. Thus, while the M0/M1/M2 signatures in the LM22-derived matrix may not best represent glioma biology, our result suggests, at minimum that a subset of macrophages exist that may play specific roles in glioma biology, including relationships with the MES-like state in IDH-WT GBM, as well as associations with 1p/19q co-deletion status in IDH-MUT glioma. An explanatory model summarizes these main interactions (Fig. 7). Based on these findings, future analyses will examine signature matrices derived from glioma single cell RNA sequencing data to further characterize the interplay of macrophages and tumor cell characteristics. Even with these limitations, our findings suggest proof-of-principal to stimulate further and more detailed studies on the role of immune cell gene expression and biology in the clinical and biological aspects of both IDH-WT and IDH-MUT diffuse gliomas.

## Conclusion

In conclusion, our analysis relies on a large sample set of gliomas to demonstrate immune cell gene expression signatures, derived by deconvolution with CIBERSORTx correlate with IDH mutation status in glioma and also with patient outcome within IDH-WT and IDH-MUT gliomas. The mesenchymal cell state in IDH-WT GBM showed association with M0 and M2 macrophages where it correlates with mesenchymal signatures in M0 macrophages, and immune signatures in M2 macrophages. Together these results highlighted the prominent association of monocytic lineage cells, specially the M0 macrophages, with MES-like state and the patient outcome thus provide insights for future investigation to better understand glioma biology and developing better immunotherapeutic approaches in gliomas.

## Supplementary Information

**Additional file 1: Figure S1**. Flowchart to represent the workflow followed in this study.

**Additional file 2: Figure S2**. **A**) Stacked bar plots representing the relative proportion of 22 immune cell types across all IDH-WT and IDH-MUT samples, where each color indicates each cell-type. **B**) Scatter plots representing a significant positive correlation between the proportion of macrophage M2 with number of segments or with fraction altered or with homologous recombination defects, where ‘‘fraction altered’’ represents the fraction of bases deviating from baseline ploidy (defined as above 0.1 or below -0.1 in log2 relative copy number (CN) space), while ‘‘number of segments’’ represents total number of segments in each sample’s copy number profile. **C**) M2 macrophage-based gene expression clusters represented by tSNE, followed by **D**) bar-plots showing IDH specific enrichment in each cluster, **E**) Kaplan–Meier curves denoting distinct survival between the clusters, and **F**) forest plots representing the survival differences corrected by IDH status, grade, and age.

**Additional file 3: Figure S3**. Gene expression clustering and the separation between IDH-MUT and IDH-WT tumors. **A**) UMAPs denoting 10 independent gene expression clusters identified in all 3 datasets harboring a clear separation between IDH-MUT (green) and IDH-WT (orange) tumors. **B)** Bar-plots showing IDH specific enrichment in each cluster, **C)** Kaplan–Meier curves denoting distinct survival between the clusters, and **D)** forest plots representing the survival differences corrected by IDH status, grade, and age.

**Additional file 4: Figure S4**. Deconvolved M2 macrophages gene expression profiles in non-glioma tumors. Unsupervised clustering of M2 macrophages gene expression profiles. **A)** tSNE plot representing the samples colored by their cluster groups. **B)** Kaplan–Meier curves estimating survival probability for each unsupervised cluster.

**Additional file 5: Figure S5**. Tumor groups with distinct immune signatures based on LM22 clusters. Heatmaps representing the hierarchical clustering of LM22 clusters. The above annotation bars representing the distributions of cluster assignments, tumor grade, MGMT promoter methylation, EGFR expression, TERT expression (surrogating the status of TERT promoter mutation) and Chr7 gain & Chr10 loss or Chr1p/19q loss with Kaplan–Meier curves below the heatmap denoting their survival differences between these immune-based clusters for **A)** IDH-WT and **B)** IDH-MUT tumors.

**Additional file 6: Figure S6**. Forest plots displaying prognostic association of the clusters from selected cell types which were consistently significant in **A)** IDH-WT tumors that involve **i)** M0 Macrophages, **ii)** M2 macrophages, **iii)** Dendritic activated cells and **iv)** B memory cells. Similarly, forest plots representing the prognostic clusters from **B)** IDH-MUT tumors which were significantly consistent across all datasets involving **i)** M0 Macrophages, **ii)** T helper and **iii)** B memory cells.

**Additional file 7: Figure S7**. Box plots demonstrating distribution of **A)** ITGA4 expression, **B)** cytolytic scores, and **C)** T cell exhaustion scores between the two immune-based survival groups.

**Additional file 8: Figure S8**. **A**) Stacked bar plots representing the relative proportion of 4 malignant cell states across all IDH-WT samples. **B)** Stacked bar plots representing the relative proportion of 3 malignant cell states in IDH-MUT tumors. **C)** Dodged boxplots depicting differential representation of 3 IDH-MUT specific malignant cell states between the two immune-based survival groups derived from 3 datasets.

**Additional file 9: Figure S9**. Scatter plots representing the correlation between proportions of **A)** M1 macrophages, **B)** M2 macrophages, and **C)** monocytes with MES-like component of all IDH-WT tumors from each dataset.

**Additional file 10: Figure S10**. **A**) UMAP representing unsupervised clustering of 89,926 cells from 19 tumors revealed 18 distinct clusters. **B**) Color-coded UMAP representing sample-wise composition of each cluster. **C**) Copy number-based identification of malignant cells and are marked in red and green. **D**) Color-coded UMAP representing distinct cell types identified from. **E**) IDH-WT tumors specific single cell derived signature matrix.

**Additional file 11: Figure S11**. Venn diagram demonstrating the number of MES-like correlating genes from **A)** M0 macrophages and **B)** M2 macrophages that were common in all 3 IDH-WT datasets followed by significantly enriched overlapping pathways.

**Additional file 12: Figure S12**. Venn diagram highlighting the number of common genes upregulated in non-codeleted tumors compared to 1p/19q codeleted IDH-MUT tumors in all 3 datasets from **A**) M0 macrophages, **B**) M2 macrophages and **C**) Tcell CD8 cells, followed by significantly enriched overlapping pathways.

**Additional file 13: Table S1**. Table listing abbreviations used for all the 22 cell types from LM22 and 10 cell types from LM10 utilized for deconvolution.

**Additional file 14: Table S2**. Table listing cluster memberships for each sample derived from cluster of cluster analysis of immune based clusters.

**Additional file 15: Table S3**. Table listing inhouse samples with scRNA Seq, with cell type annotation and scRNA derived signature matrix.
