# Supplementary figures and images for: Immune cell gene expression signatures in diffuse glioma are associated with IDH mutation status, patient outcome and malignant cell state, and highlight the importance of specific cell subsets in glioma biology
Source: Acta Neuropathol Commun. 2022 Feb 10;10:19. doi: 10.1186/s40478-022-01323-w (PMC8830123; doi:10.1186/s40478-022-01323-w)

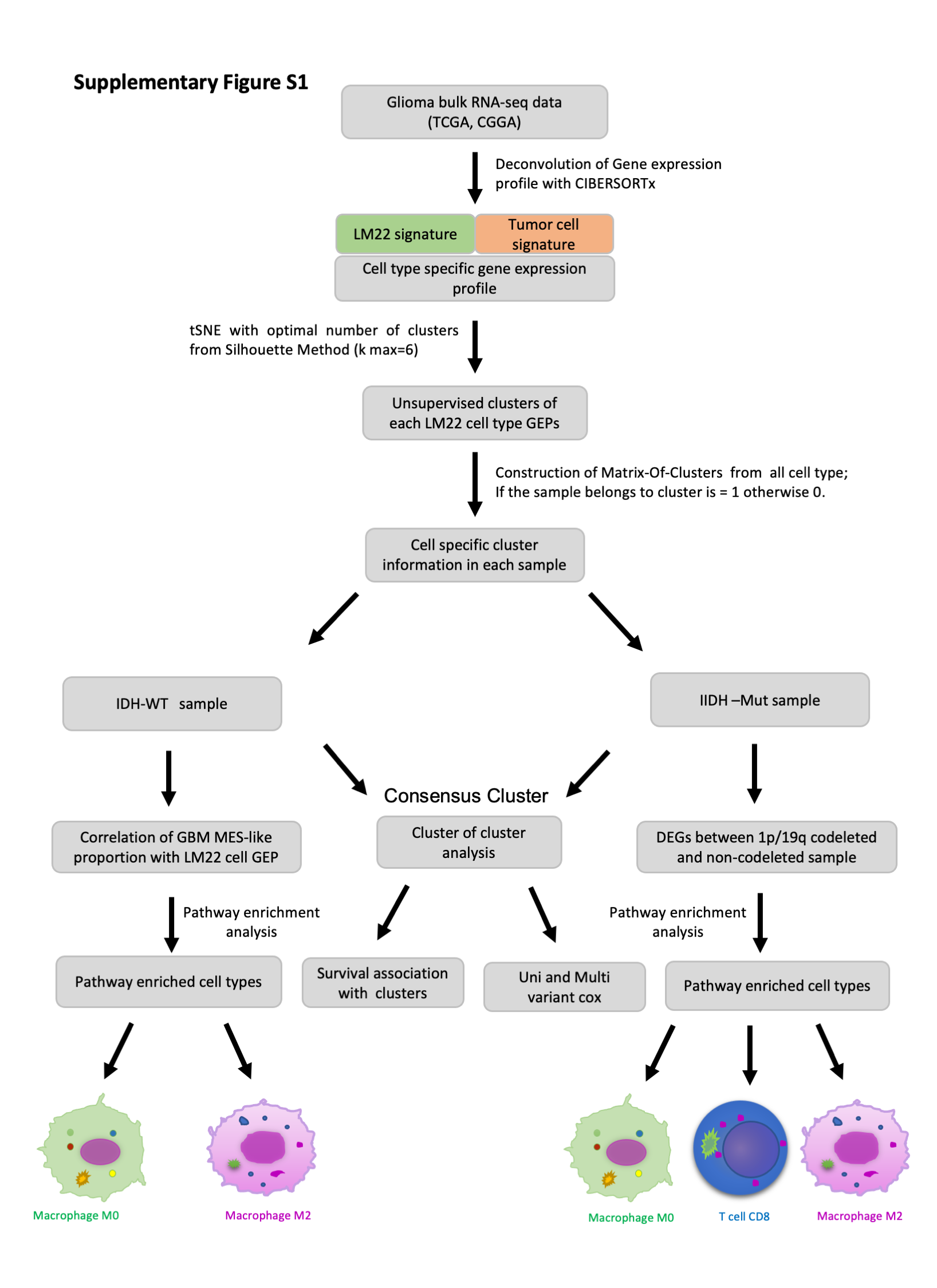

Supplement: Supplementary file 1 — Additional file 1: Figure S1. Flowchart to represent the workflow followed in this study. [file 40478_2022_1323_MOESM1_ESM.tiff]

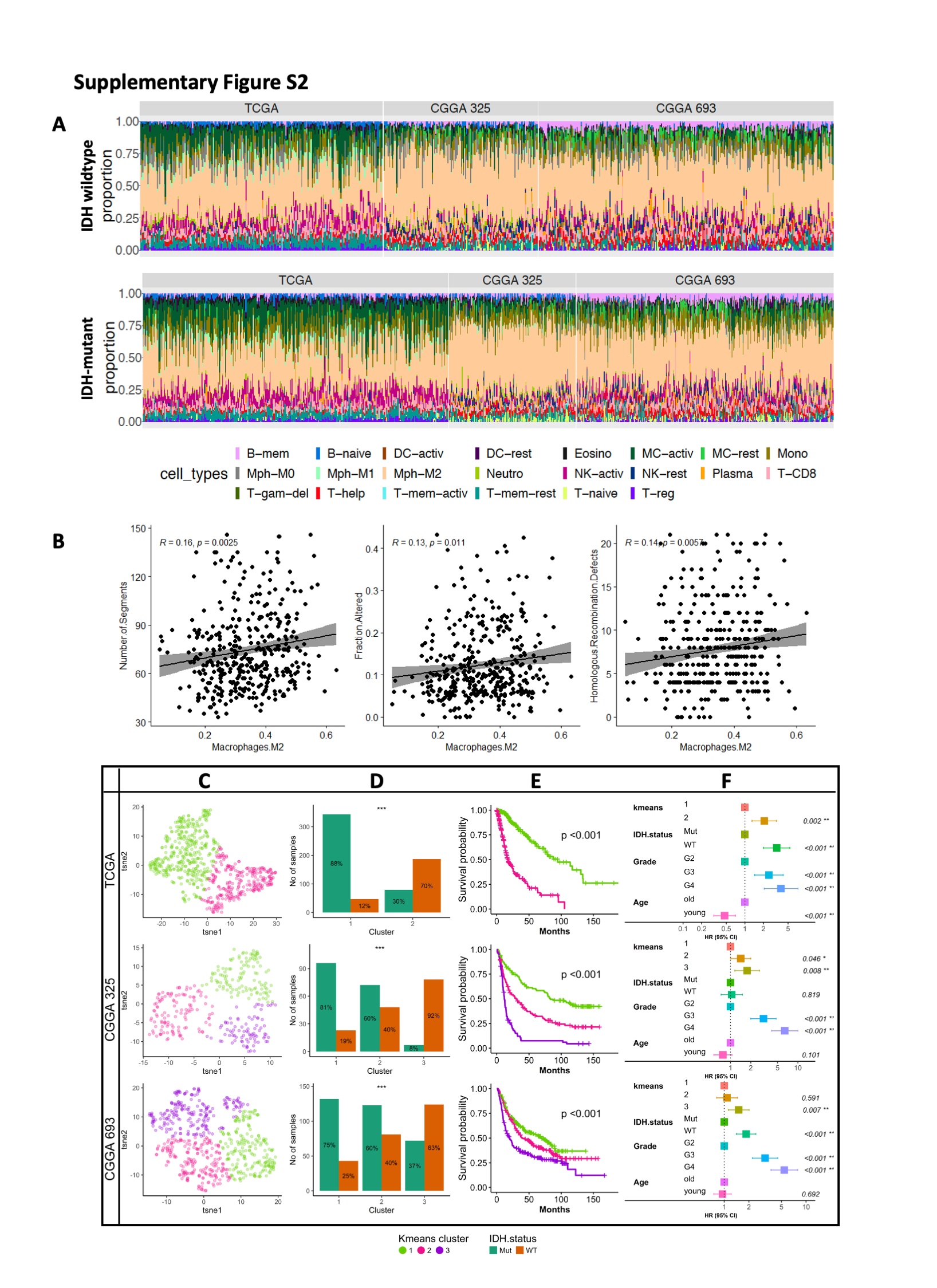

Supplement: Supplementary file 2 — Additional file 2: Figure S2. A) Stacked bar plots representing the relative proportion of 22 immune cell types across all IDH-WT and IDH-MUT samples, where each color indicates each cell-type. B) Scatter plots representing a significant positive correlation between the proportion of macrophage M2 with number of segments or with fraction altered or with homologous recombination defects, where ‘‘fraction altered’’ represents the fraction of bases deviating from baseline ploidy (defined as above 0.1 or below -0.1 in log2 relative copy number (CN) space), while ‘‘number of segments’’ represents total number of segments in each sample’s copy number profile. C) M2 macrophage-based gene expression clusters represented by tSNE, followed by D) bar-plots showing IDH specific enrichment in each cluster, E) Kaplan–Meier curves denoting distinct survival between the clusters, and F) forest plots representing the survival differences corrected by IDH status, grade, and age. [file 40478_2022_1323_MOESM2_ESM.tiff]

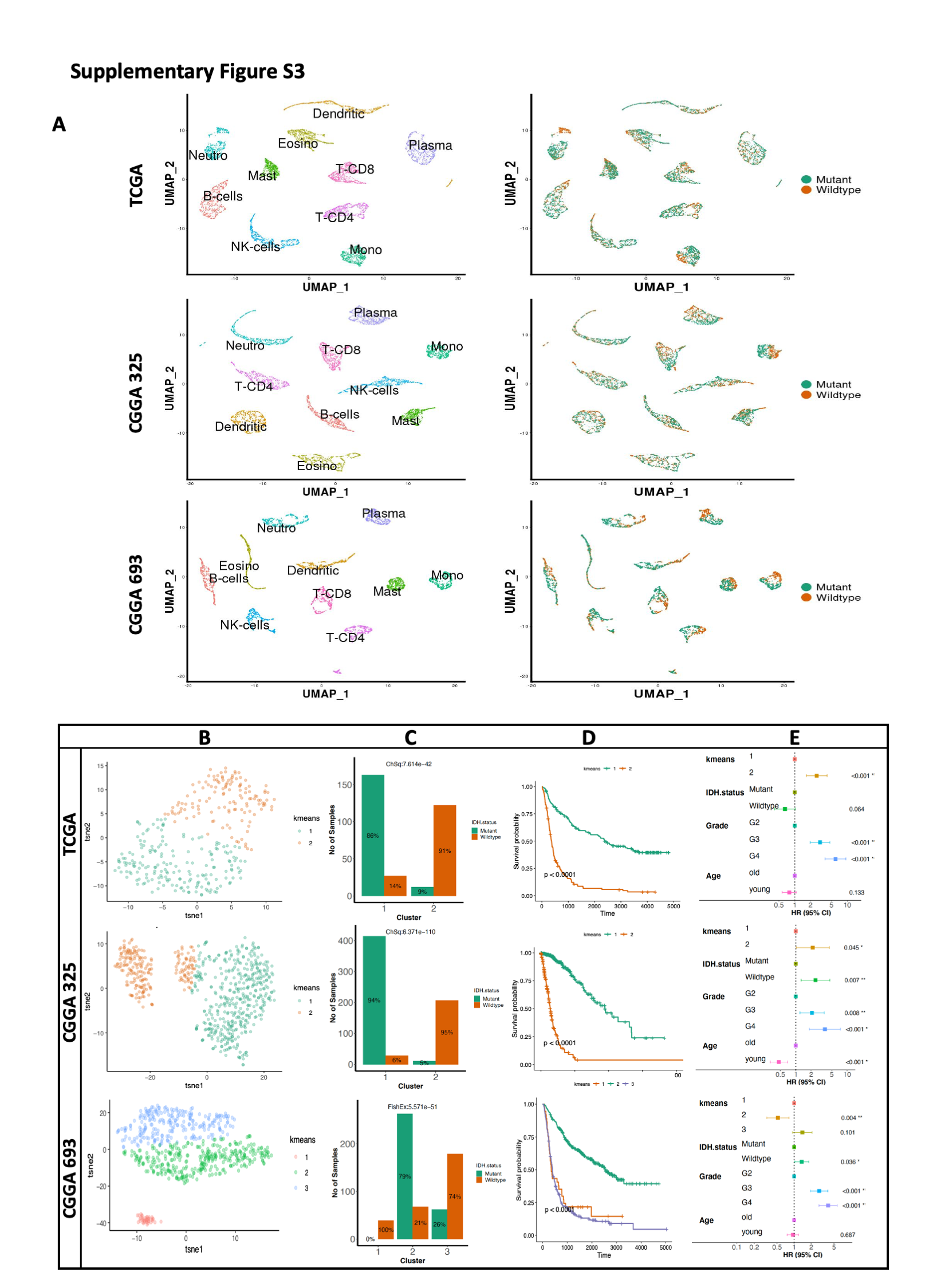

Supplement: Supplementary file 3 — Additional file 3: Figure S3. Gene expression clustering and the separation between IDH-MUT and IDH-WT tumors. A) UMAPs denoting 10 independent gene expression clusters identified in all 3 datasets harboring a clear separation between IDH-MUT (green) and IDH-WT (orange) tumors. B) Bar-plots showing IDH specific enrichment in each cluster, C) Kaplan–Meier curves denoting distinct survival between the clusters, and D) forest plots representing the survival differences corrected by IDH status, grade, and age. [file 40478_2022_1323_MOESM3_ESM.tiff]

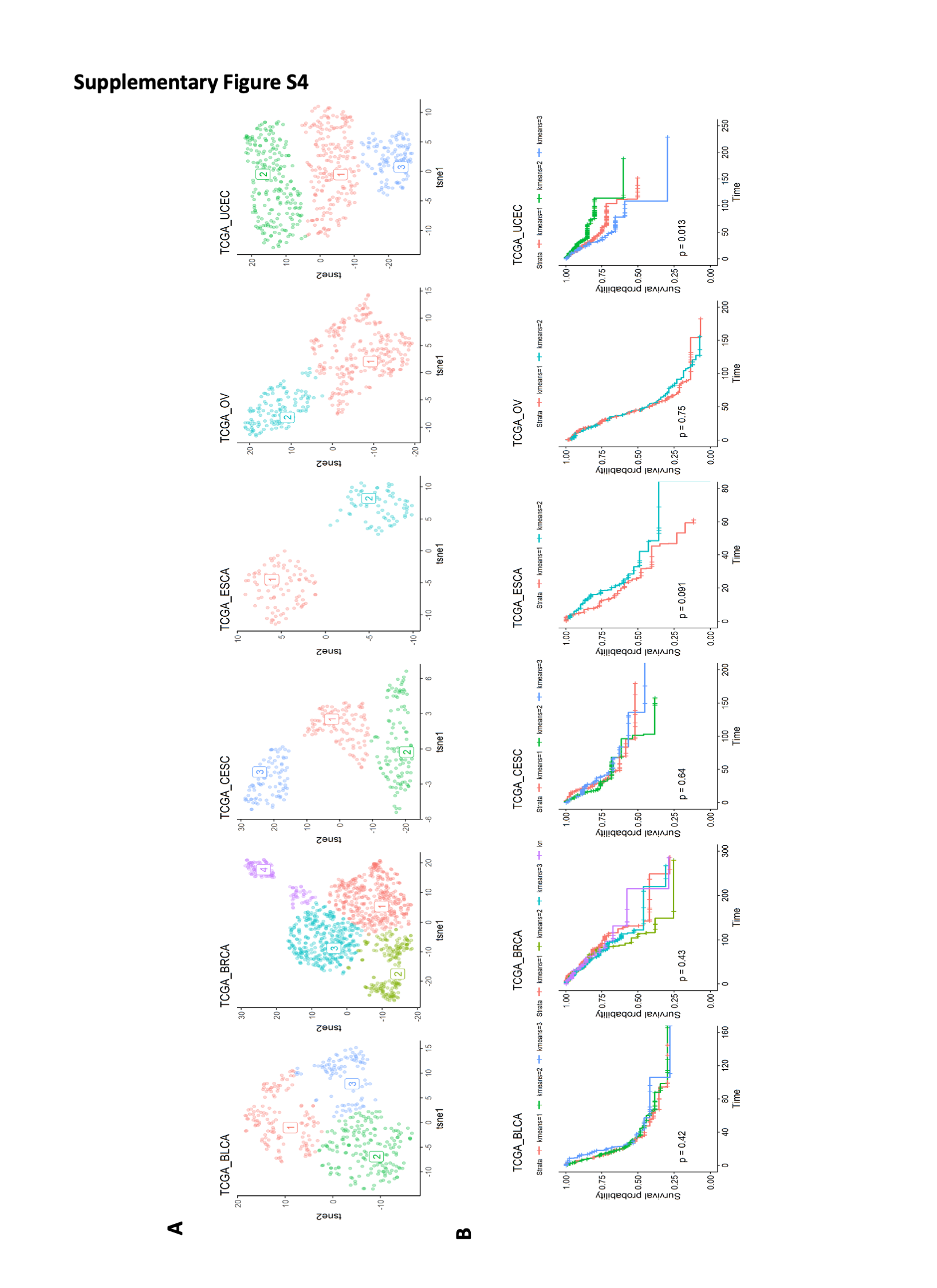

Supplement: Supplementary file 4 — Additional file 4: Figure S4. Deconvolved M2 macrophages gene expression profiles in non-glioma tumors. Unsupervised clustering of M2 macrophages gene expression profiles. A) tSNE plot representing the samples colored by their cluster groups. B) Kaplan–Meier curves estimating survival probability for each unsupervised cluster. [file 40478_2022_1323_MOESM4_ESM.tiff]

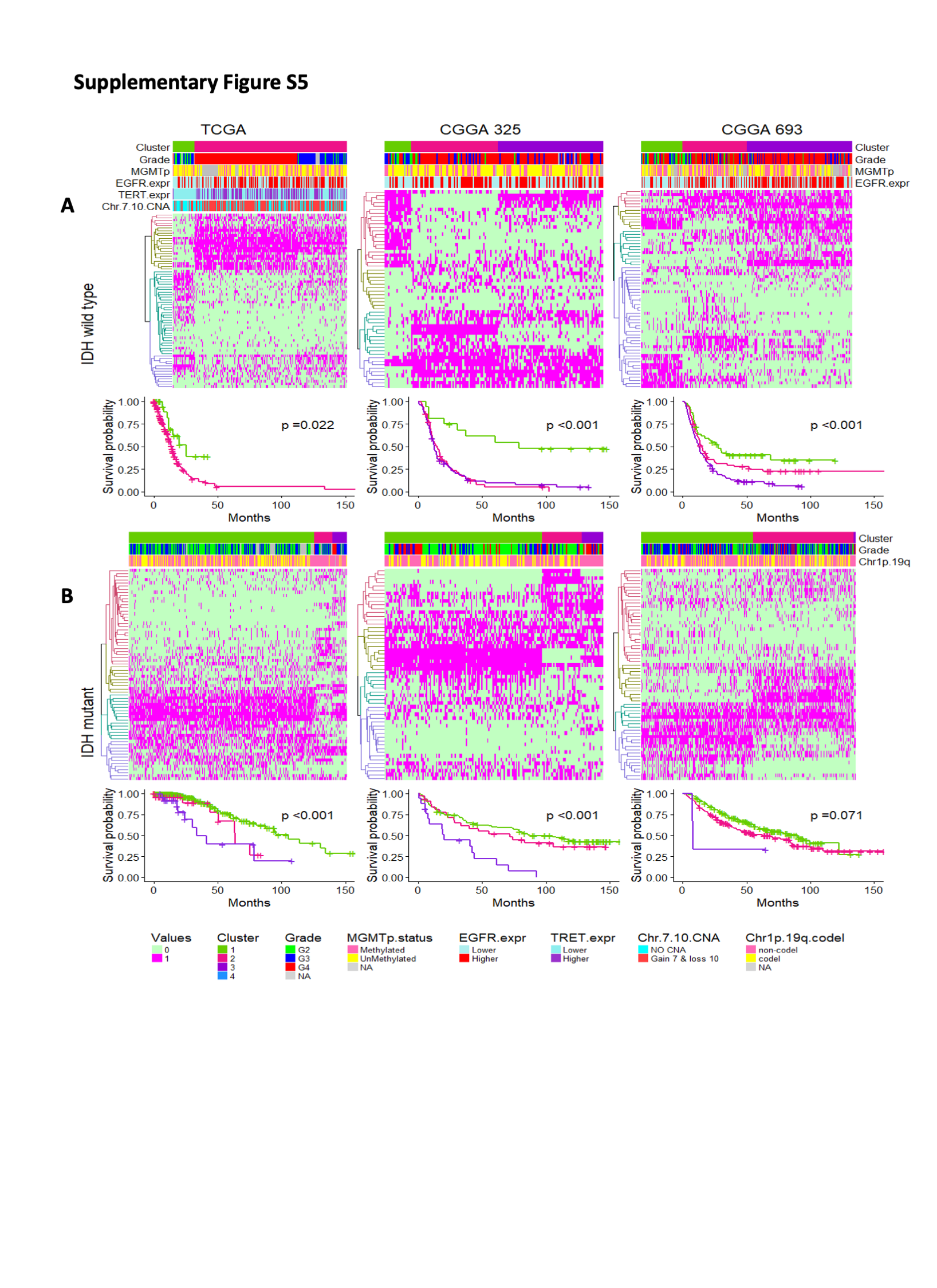

Supplement: Supplementary file 5 — Additional file 5: Figure S5. Tumor groups with distinct immune signatures based on LM22 clusters. Heatmaps representing the hierarchical clustering of LM22 clusters. The above annotation bars representing the distributions of cluster assignments, tumor grade, MGMT promoter methylation, EGFR expression, TERT expression (surrogating the status of TERT promoter mutation) and Chr7 gain & Chr10 loss or Chr1p/19q loss with Kaplan–Meier curves below the heatmap denoting their survival differences between these immune-based clusters for A) IDH-WT and B) IDH-MUT tumors. [file 40478_2022_1323_MOESM5_ESM.tiff]

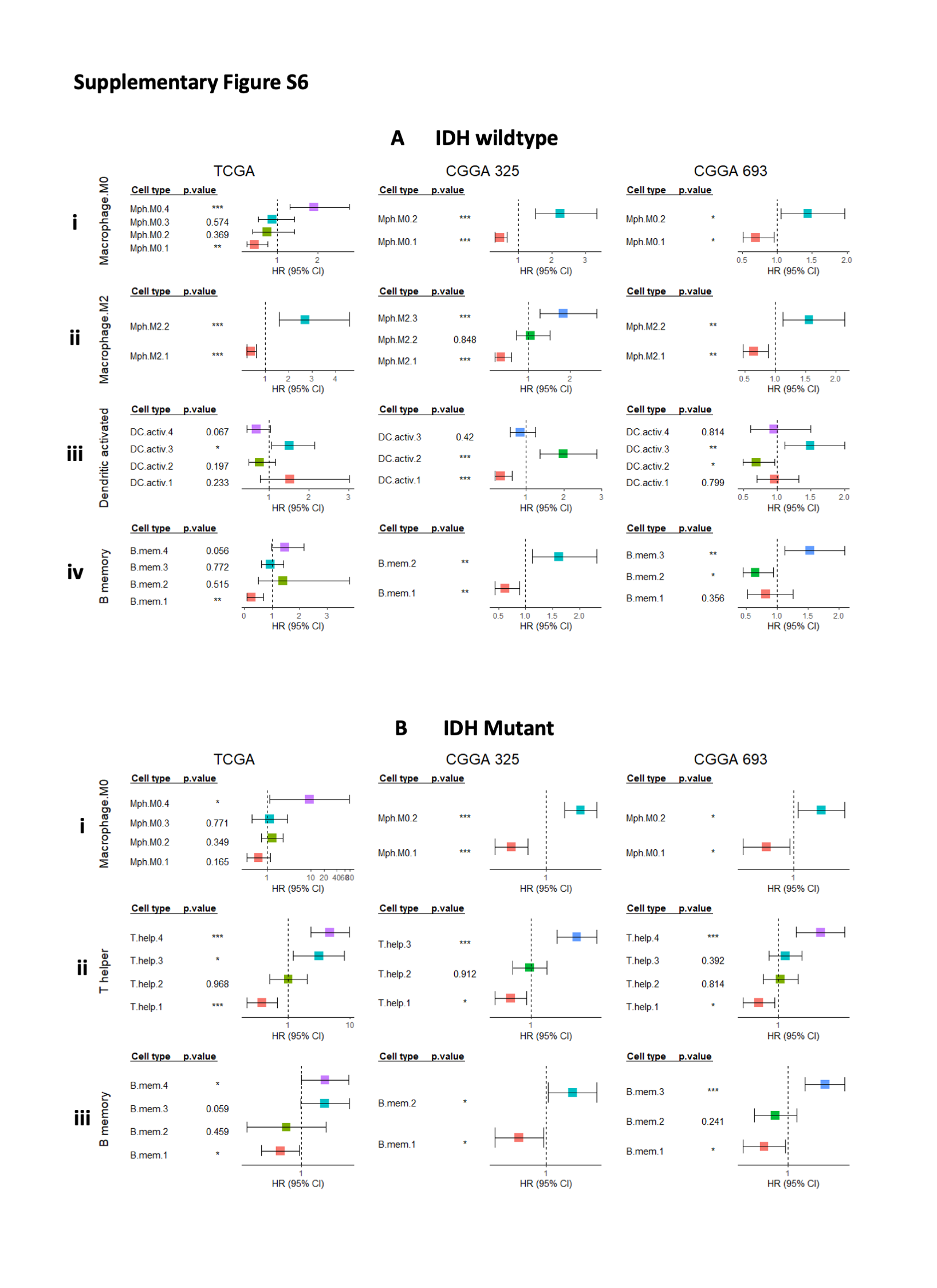

Supplement: Supplementary file 6 — Additional file 6: Figure S6. Forest plots displaying prognostic association of the clusters from selected cell types which were consistently significant in A) IDH-WT tumors that involve i) M0 Macrophages, ii) M2 macrophages, iii) Dendritic activated cells and iv) B memory cells. Similarly, forest plots representing the prognostic clusters from B) IDH-MUT tumors which were significantly consistent across all datasets involving i) M0 Macrophages, ii) T helper and iii) B memory cells. [file 40478_2022_1323_MOESM6_ESM.tiff]

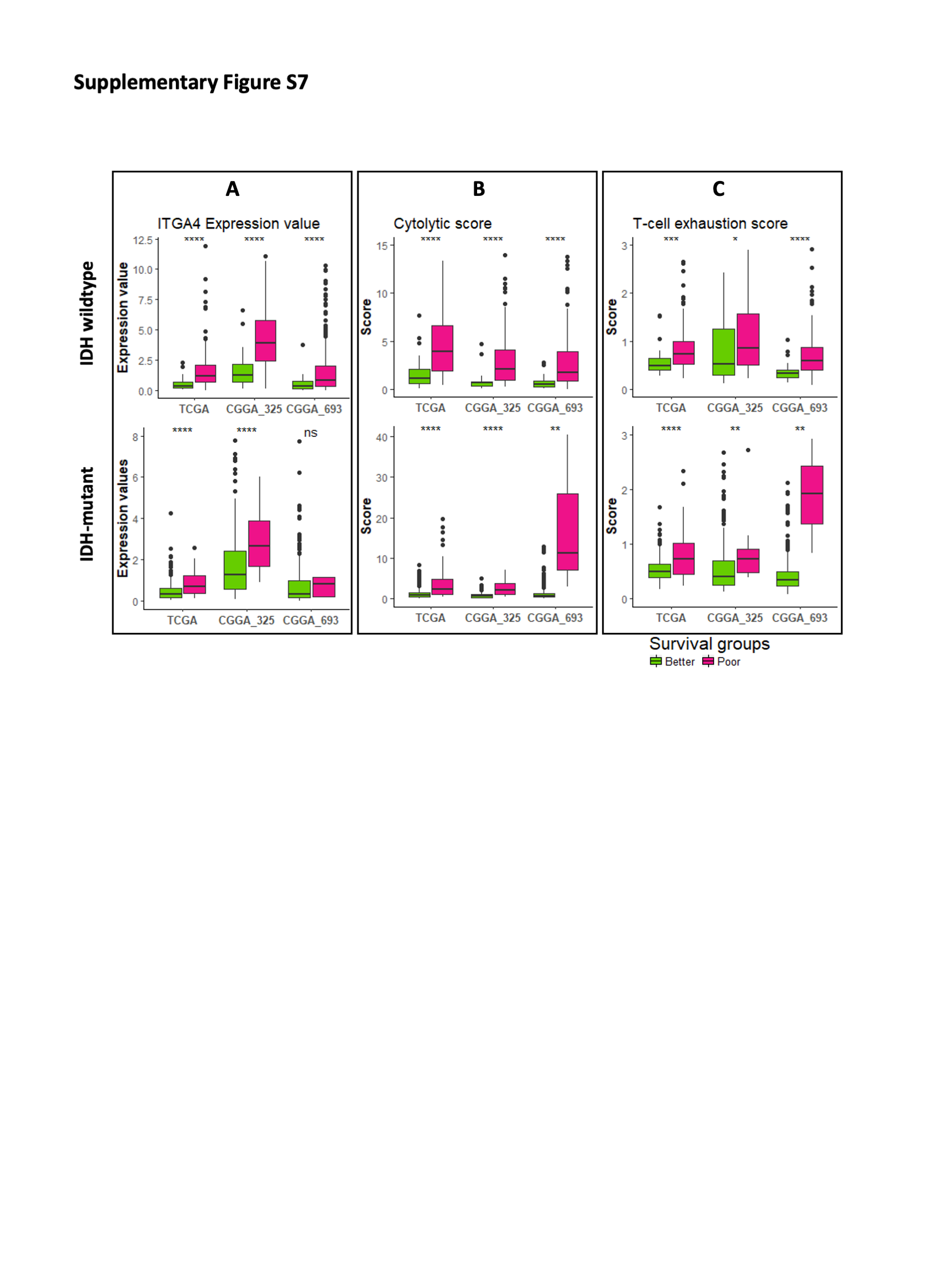

Supplement: Supplementary file 7 — Additional file 7: Figure S7. Box plots demonstrating distribution of A) ITGA4 expression, B) cytolytic scores, and C) T cell exhaustion scores between the two immune-based survival groups. [file 40478_2022_1323_MOESM7_ESM.tiff]

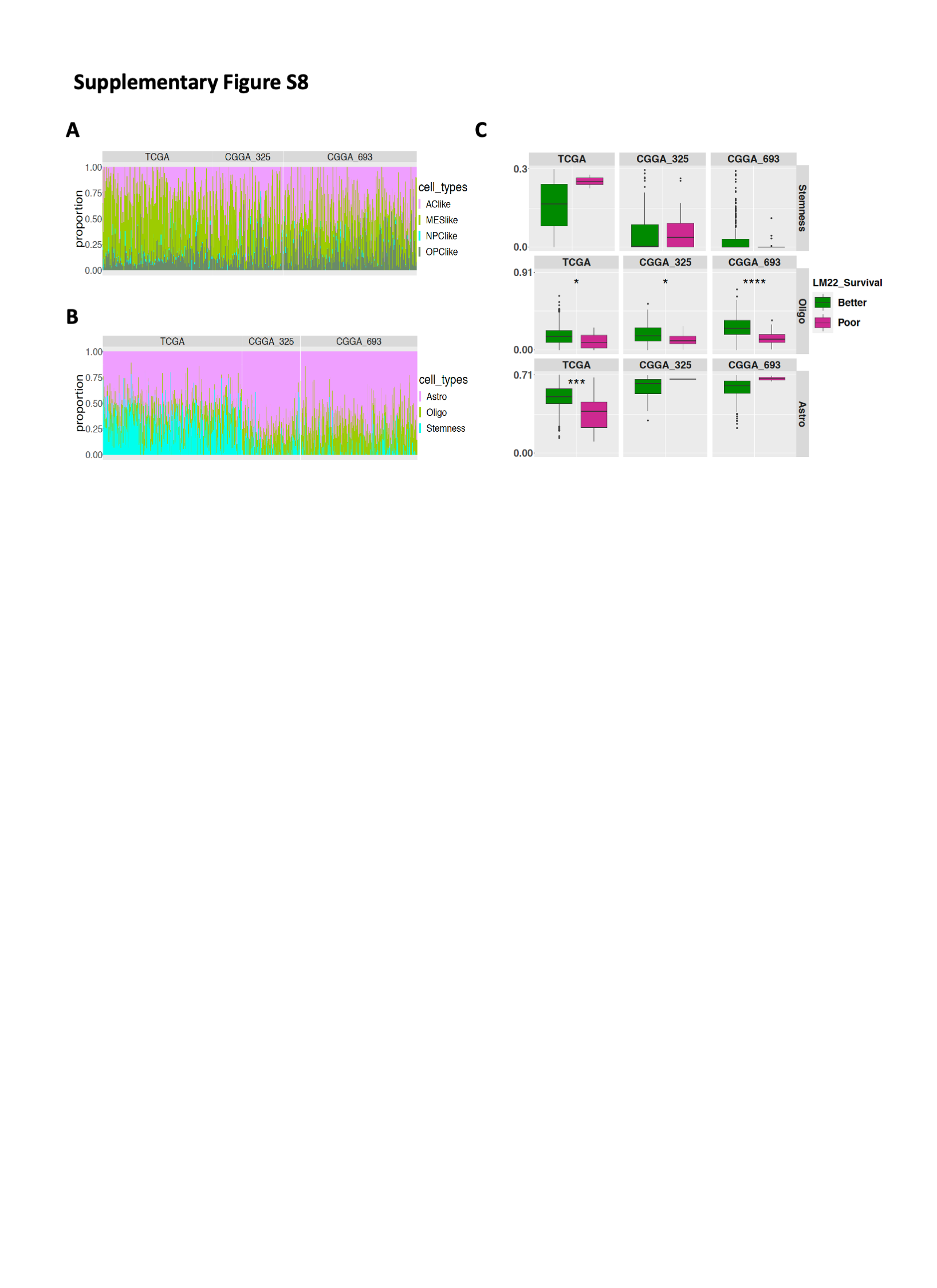

Supplement: Supplementary file 8 — Additional file 8: Figure S8. A) Stacked bar plots representing the relative proportion of 4 malignant cell states across all IDH-WT samples. B) Stacked bar plots representing the relative proportion of 3 malignant cell states in IDH-MUT tumors. C) Dodged boxplots depicting differential representation of 3 IDH-MUT specific malignant cell states between the two immune-based survival groups derived from 3 datasets. [file 40478_2022_1323_MOESM8_ESM.tiff]

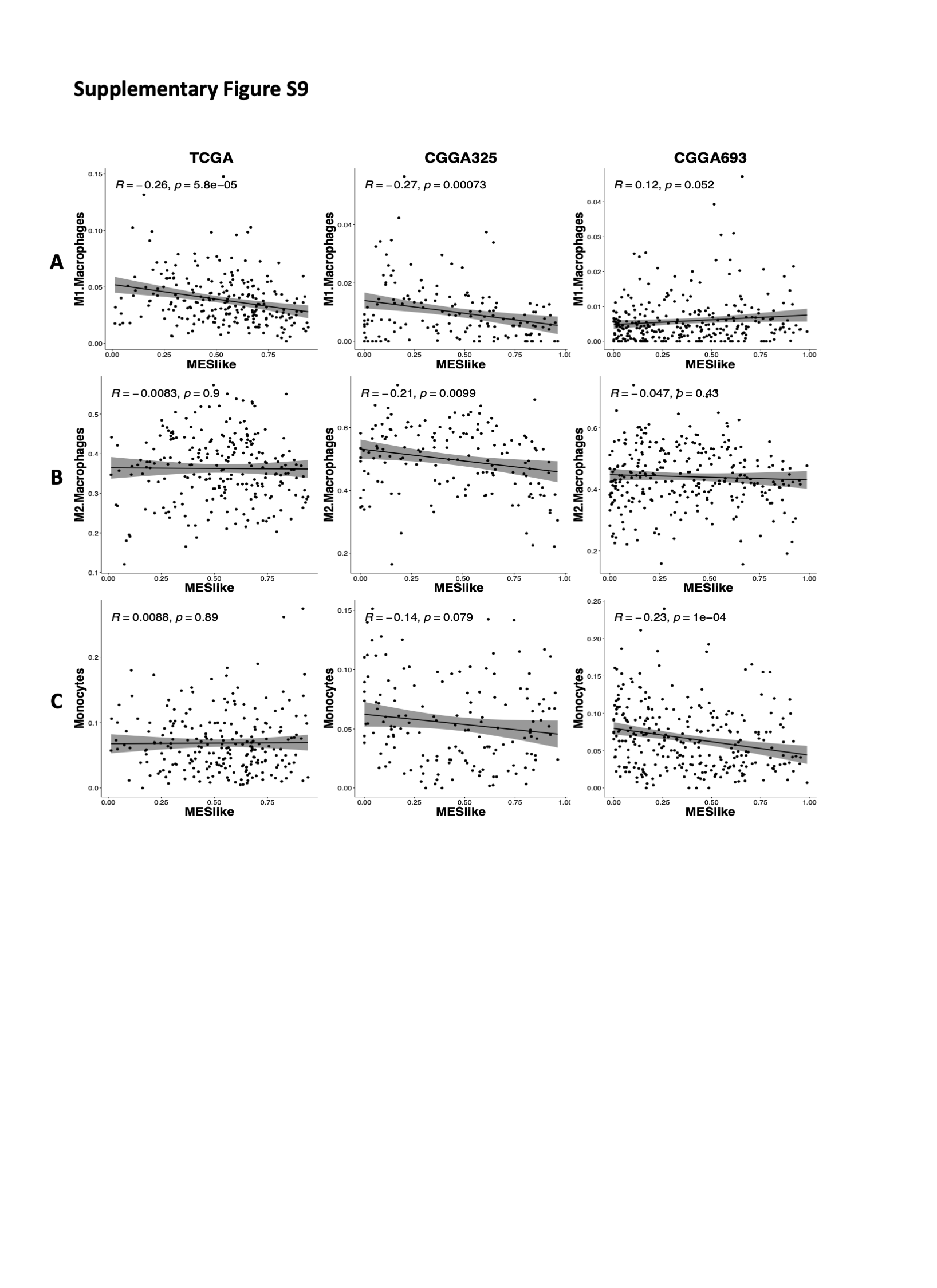

Supplement: Supplementary file 9 — Additional file 9: Figure S9. Scatter plots representing the correlation between proportions of A) M1 macrophages, B) M2 macrophages, and C) monocytes with MES-like component of all IDH-WT tumors from each dataset. [file 40478_2022_1323_MOESM9_ESM.tiff]

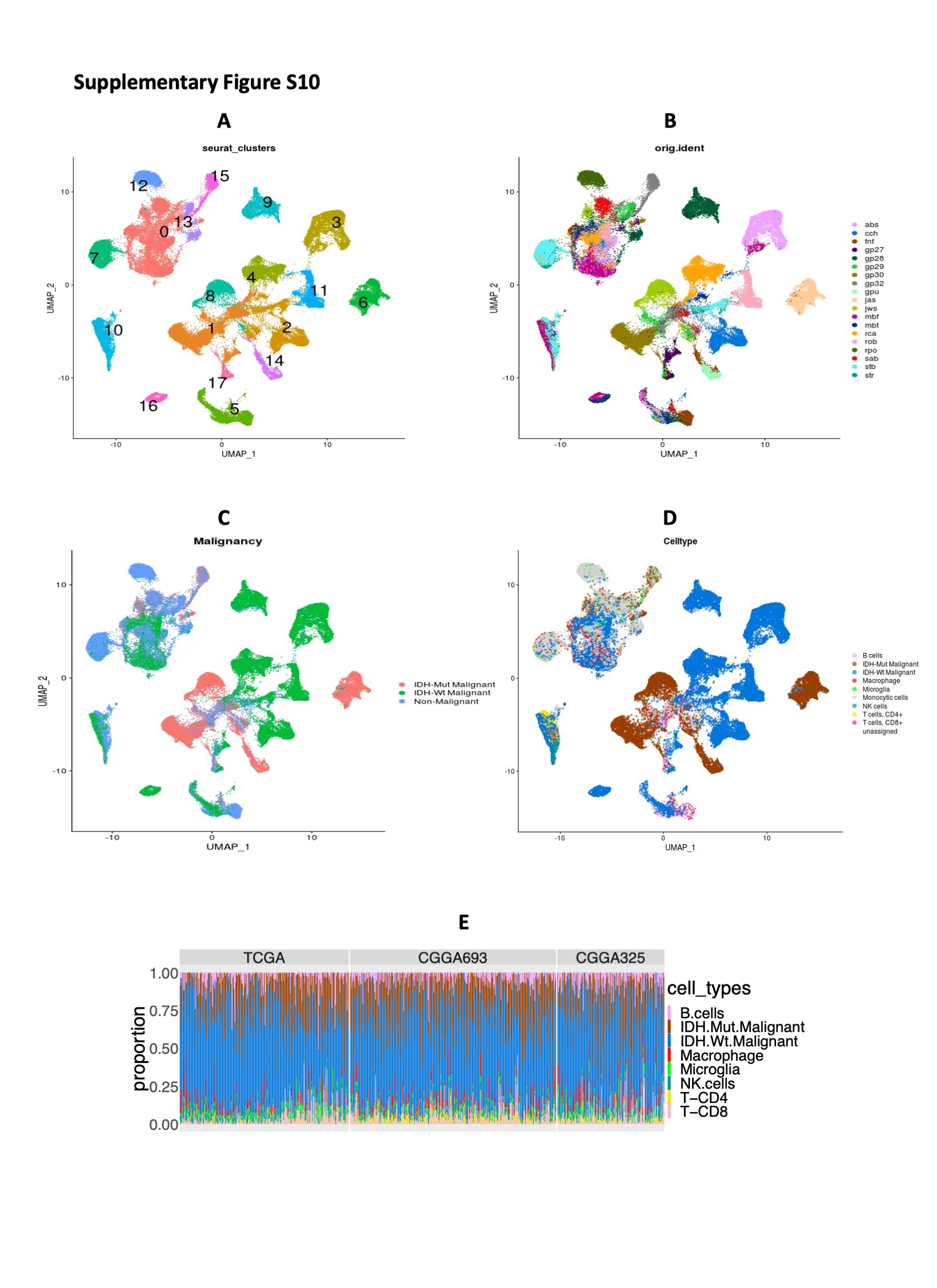

Supplement: Supplementary file 10 — Additional file 10: Figure S10. A) UMAP representing unsupervised clustering of 89,926 cells from 19 tumors revealed 18 distinct clusters. B) Color-coded UMAP representing sample-wise composition of each cluster. C) Copy number-based identification of malignant cells and are marked in red and green. D) Color-coded UMAP representing distinct cell types identified from. E) IDH-WT tumors specific single cell derived signature matrix. [file 40478_2022_1323_MOESM10_ESM.tiff]

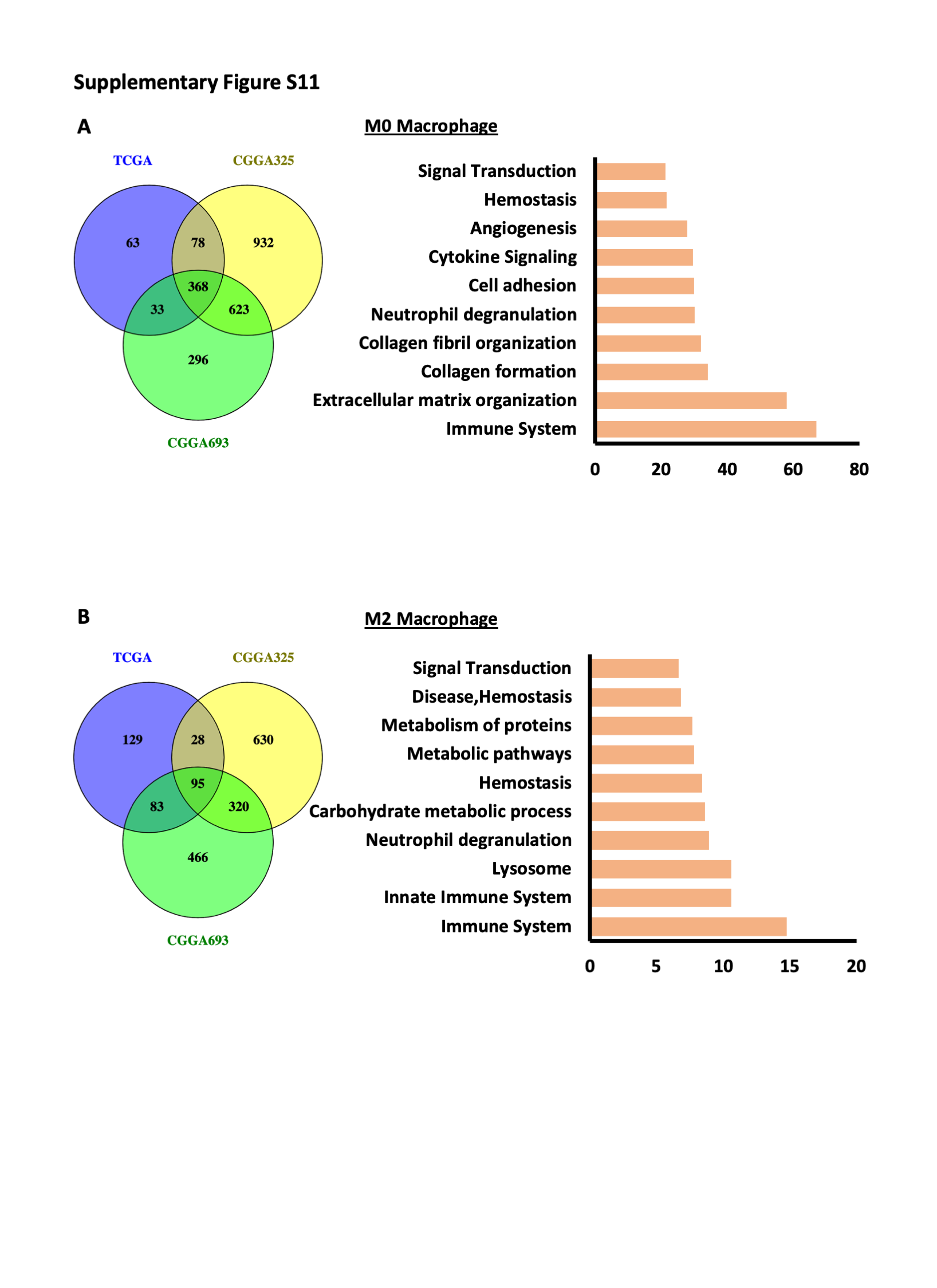

Supplement: Supplementary file 11 — Additional file 11: Figure S11. Venn diagram demonstrating the number of MES-like correlating genes from A) M0 macrophages and B) M2 macrophages that were common in all 3 IDH-WT datasets followed by significantly enriched overlapping pathways. [file 40478_2022_1323_MOESM11_ESM.tiff]

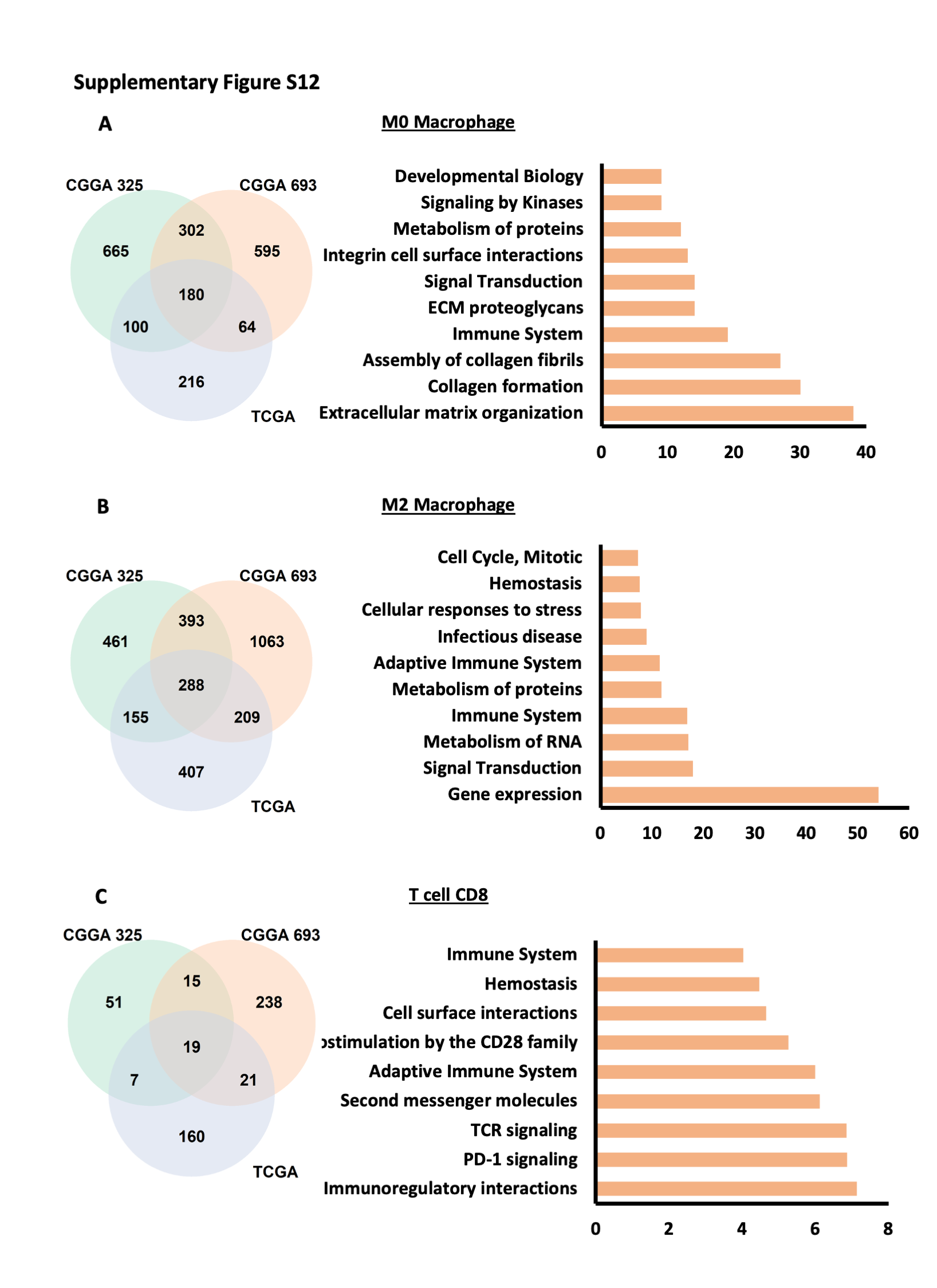

Supplement: Supplementary file 12 — Additional file 12: Figure S12. Venn diagram highlighting the number of common genes upregulated in non-codeleted tumors compared to 1p/19q codeleted IDH-MUT tumors in all 3 datasets from A) M0 macrophages, B) M2 macrophages and C) Tcell CD8 cells, followed by significantly enriched overlapping pathways. [file 40478_2022_1323_MOESM12_ESM.tiff]
